# Continuity of Short-Time Dynamics Crossing the Liquid–Liquid Phase Separation in Charge-Tuned Protein Solutions

**DOI:** 10.1021/acs.jpclett.4c02533

**Published:** 2024-11-26

**Authors:** Ilaria Mosca, Christian Beck, Niina H. Jalarvo, Olga Matsarskaia, Felix Roosen-Runge, Frank Schreiber, Tilo Seydel

**Affiliations:** † Institut für Angewandte Physik, Universität Tübingen, Auf der Morgenstelle 10, 72076 Tübingen, Germany; ‡ Institut Max von Laue−Paul Langevin, 71 Av. des Martyrs, 38042 Grenoble, France; § Neutron Scattering Division, Oak Ridge National Laboratory, 5200, 1 Bethel Valley Rd, Oak Ridge, Tennessee 37830, United States; ∥ Division of Physical Chemistry, Lund University, Naturvetarvägen 14, 22362 Lund, Sweden

## Abstract

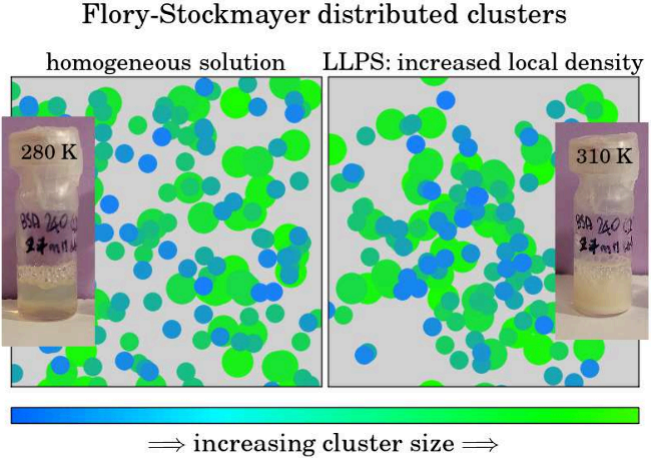

Liquid–liquid phase separation (LLPS) constitutes a crucial phenomenon in biological self-organization, not only intervening in the formation of membraneless organelles but also triggering pathological protein aggregation, which is a hallmark in neurodegenerative diseases. Employing incoherent quasi-elastic neutron spectroscopy (QENS), we examine the short-time self-diffusion of a model protein undergoing LLPS as a function of phase splitting and temperature to access information on the nanosecond hydrodynamic response to the cluster formation both within and outside the LLPS regime. We investigate the samples as they dissociate into microdroplets of a dense protein phase dispersed in a dilute phase as well as the separated dense and dilute phases obtained from centrifugation. By interpreting the QENS results in terms of the local concentrations in the two phases determined by UV–vis spectroscopy, we hypothesize that the short-time transient protein cluster size distribution is conserved at the transition point while the local volume fractions separate.

Metastable liquid–liquid phase separation (LLPS) of protein solutions plays crucial roles in protein crystallization,^1^ cellular signaling,^2^ pathologies such as eye cataracts,^3^ and metabolic^4,5^ and neurodegenerative diseases.^6,7^ LLPS is increasingly being understood as a fundamental mechanism of biological self-organization, self-assembly,^8−15^ and functional regulation.^16−18^ Moreover, the naturally occurring LLPS inspires an interest in synthetically employing this phenomenon.^19^ The understanding of protein–protein interactions and the underlying phase behavior is thus of outstanding importance in, for example, healthcare and pharmaceutical applications, as well as in bioengineering and nanotechnology. LLPS is a thermodynamically driven process in which a homogeneous protein solution splits into micro- or nanodroplets of a protein-rich phase immersed in a dilute phase. The two coexisting phases are maintained through balanced chemical potentials of solutes and osmotic potentials of solvents, arising from the minimization of the total free energy.^20,21^ This separation has been described by random phase approximation theories enhanced by Flory–Huggins interaction models^22,23^ for the case of intrinsically disordered proteins (IDPs) that constitute the majority of proteins for which LLPS has been found.^23,24^ LLPS has also been described as a dynamic outcome of the interplay between a diffusion-limited protein–protein encounter and the exhaustion of available valencies within smaller clusters^25^ and has been discussed in terms of a phase transition.^26−28^

Thermodynamically, the demixing of an initially homogeneous protein solution is associated with a dramatic increase in correlation length visible by small-angle scattering^29^ as well as by photon correlation spectroscopy,^30^ possibly preceded by a bimodal cluster size distribution.^31^ Other models for LLPS in globular proteins include adhesive hard-sphere models to describe dynamical arrest, involving percolating clusters.^32−34^ Moreover, patchy-particle models^35,36^ as well as simulations^37^ have been employed to predict phase diagrams. Picosecond time-resolved fluorescence anisotropy showed that nanosecond transient nanoclusters underlie the long-range correlations in protein LLPS.^38^ Using static and dynamic light scattering (SLS/DLS), a universal osmotic equation of state and dynamical behavior were previously observed for lysozyme protein solutions undergoing LLPS.^39^ It was discussed in terms of the theory of corresponding states^39−43^ that close to LLPS, the osmotic compressibility and the collective diffusion of protein solutions are only determined by the volume fraction and the temperature relative to the critical temperature or, equivalently, by the second virial coefficient,^39^ and that the sensitivity of thermodynamic properties to the details of the underlying interactions vanishes.^39^

In terms of colloid theory,^2^ metastable LLPS is driven by a short-range attraction between the colloid particles. This concept can also be applied to proteins, for instance, by employing trivalent metal cations, e.g., Y^3+^, Ho^3+^, and La^3+^, to create this short-range attraction for proteins such as bovine serum albumin (BSA).^44^ The multivalent metal ions provide an efficient and ion-specific way to tune interactions in protein solutions,^45−47^ where binding and bridging of the metal ions lead to a rich phase behavior that includes crystallization, re-entrant condensation, and metastable LLPS with a lower critical solution temperature (LCST-LLPS)^44,48^ (Figure 1). Protein cluster formation has been shown and characterized for aqueous solutions of BSA and multivalent salts,^47,49^ including microscopy^47,50,51^ and light scattering.^52^ These solutions constitute a well-investigated model system for LLPS at suitable salt concentrations,^47,49,53^ and their kinetics have been studied, for instance, by (ultra-)small-angle scattering^54^ and by NMR with trifluoroethanol as a probe of the local densities in the two phases.^55^ Theoretically, the phase behavior of BSA solutions can be understood in terms of the above-mentioned patchy colloid models.^35,49,56,57^ BSA solutions with YCl_3_ were also explored by DLS in the low-turbidity and low-concentration range accessible by this method, observing two diffusive time scales in the long-time limit of collective diffusion.^52^ Both diffusion coefficients decreased monotonously with increasing salt concentration approaching the precipitation regime that precedes LLPS.^52^ Due to the above-quoted substantial amount of previous work on BSA solutions, the BSA-LaCl_3_ system constitutes our model of choice to experimentally access the unique dynamic regime of short-time self-diffusion during LLPS by high-resolution spin-incoherent quasi-elastic neutron spectroscopy (QENS), as motivated in the following paragraph. The observation or coherence time of our experiment is on the order of 1 ns, resulting from its high energy resolution of a few μeV.

**Figure 1:**
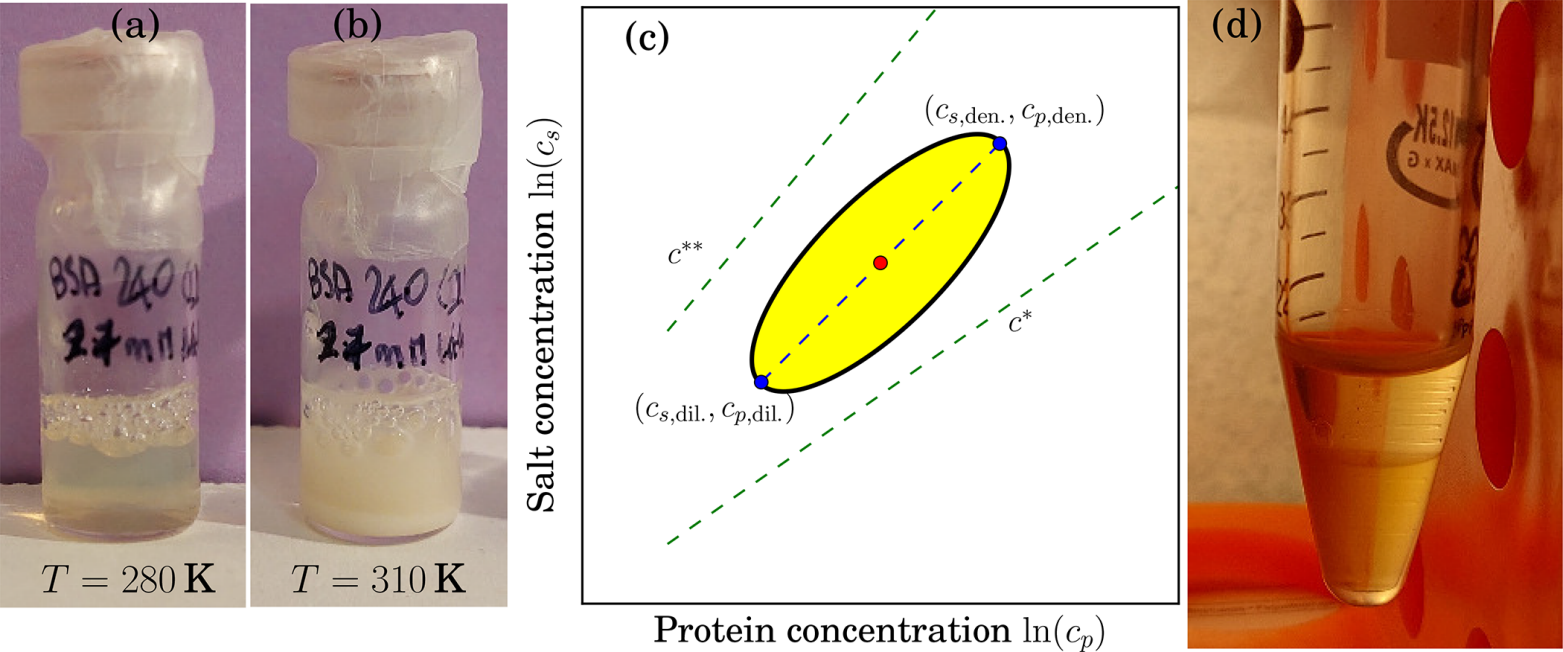
(a) BSA at *c*_p_ = 240 mg/mL with *c*_s_ = 27 mM LaCl_3_ (*c*_s_/*c*_p_ = 7.5) in D_2_O displays signatures of LLPS, being transparent at *T* ≈ 280 K and (b) milky/turbid at *T* ≈ 310 K. We note that the optical transparency alone is not a suitable criterion for LLPS, cf. main text. (c) Schematic of a cut at constant *T* through a generic protein–salt phase diagram, illustrating the splitting of a mixture prepared at the (*c*_s_, *c*_p_) point (red circle symbol) in the center of the LLPS region (shaded area). Due to LLPS, the two phases after the separation are located at the two points marked by blue circle symbols on opposite sides of the shaded LLPS region. The dashed lines labeled c* and c**, respectively, denote the salt concentration boundaries of the optically turbid regime c* < *c*_s_ < c**. (d) BSA at *c*_p_ = 240 mg/mL, *c*_s_ = 27 mM LaCl_3_ (*c*_s_/*c*_p_ = 7.5) after centrifugation for more than 12 h at 40 °C. The splitting into two well-separated phases becomes visible: the dense protein-rich phase at the bottom of the Falcon tube and the dilute protein-poor phase above. The two phases were extracted and measured separately.

Molecular dynamics (MD) simulations show that relaxation in suspensions of associative proteins slows dramatically when the stoichiometry is balanced,^58^ revealing the substantial reduction of the long-time diffusion with increasing protein–protein bond relaxation time.^58^ Since long-time diffusion is the result of both short-time hydrodynamic and longer-time direct interactions, accurate knowledge of the short-time diffusion is required to quantify the long-time diffusion. Moreover, it is essential to understand bond fluctuations and the resulting scaling behavior of diffusion described by mean-field theory,^59^ predicting a rather loose fluctuating network liquid as opposed to dynamically slowed clusters in the dense phase of LLPS.^59^ Large-scale MD simulations also reveal a highly dynamic picture of LLPS even in the dense phase,^60^ finding time scales of less than 1 μs for protein–protein partner exchange and nanosecond contact dynamics in a model of IDPs.^60^ The simulations give rise to the hypothesis that fast nanosecond diffusion even in the dense phase allows for transport and signal transduction, which are considered crucial for biological function. On this nanometer length scale associated with nanosecond diffusion, hydrodynamic and electrostatic interactions dominate over negligible direct interactions and constitute part of the picture describing possibly transient cluster formation in short-range attraction–long-range repulsion (SALR) systems.^36,61^ Notably, by measuring self-diffusion, our QENS experiment unambiguously accesses the hydrodynamic size of protein clusters unaffected by a superimposed static structure factor and, by measuring on the nanosecond time scale, detects even short-lived clusters. The nanosecond coherence time, along with the access to self-diffusion by our experiment as opposed to long-time collective diffusion accessed, for example, by DLS or long-time self-diffusion accessed, for example, by NMR, is crucial to interpret our results.

The protein solution samples were prepared following previously established protocols^62−64^ at the nominal protein concentration *c*_p_ ≔ *m*_p_/*V*_D_2_O_ = 240 mg/mL BSA in D_2_O, with the protein mass *m*_p_ and D_2_O volume *V*_D_2_O_, corresponding to the dry protein volume fraction φ = *m*_p_ν_p_/(*V*_D_2_O_ + *m*_p_ν_p_) = 0.15 with the protein specific volume ν_p_ = 0.735 mL/g,^65^ at different LaCl_3_ salt concentrations *c*_s_ = 0, 20, 23, 25, 26, 27, 29, 30, and 35 mM, corresponding to the number of salt ions per protein *c*_s_/*c*_p_ = 0.0, 5.5, 6.4, 6.9, 7.5, and 8.0, respectively. Importantly, we measured these samples in the mixed state and, at suitable salt concentrations, on samples that were separated into the dense and dilute phases by centrifuging, and we determined concentrations by UV–vis (cf. Experimental Methods).

The observable in QENS is the dynamic structure factor *S*(*q*, ω) as a function of energy transfer ℏω and momentum transfer ℏ*q*. For our samples, *S*(*q*, ω) arises from the protein and aqueous (D_2_O) solvent contributions. For globular proteins, the protein signal can be separated into the contributions from the apparent global center-of-mass (COM) diffusion of the protein or protein cluster *S*_global_(*q*, ω) and from the internal diffusion *S*_internal_(*q*, ω). The superposition of these contributions is convoluted with the spectrometer resolution function *R*(*q*, ω) obtained from a Vanadium spectrum and represented analytically by a sum of noncentered Gaussian functions^66^ (Figure S10). The measured *S*(*q*, ω) is therefore described by
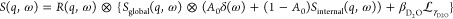
1where *A*_0_(*q*) is a scalar identified with the elastic incoherent structure factor (EISF) characterizing dynamic confinement effects^67^ and δ(ω) is the Dirac function. The scalar β_D_2_O_(*q*) is fixed in the fits based on a separate pure solvent measurement, accounting for the volume excluded by BSA, and the D_2_O signal is modeled by one Lorentzian  with the width γ_D_2_O_(*q*) fixed from existing neutron time-of-flight data.^68,69^ For BSA in solution, it has been shown that the internal diffusive processes of the backbone and the side chains accounted for by *S*_internal_ can be separated and described by two coupled Lorentzians on the accessible ℏω range of BASIS^64,70,71^ (cf. Experimental Methods, eqs 6 and 7). It has also been shown that the clusters, which for BSA–trivalent salt solutions are distributed according to the Flory–Stockmayer theory,^35,63,70,72,73^ can be represented by a single Lorentzian  with width γ(*q*) accounting for the effective cluster COM diffusion:
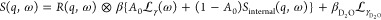
2Therein, we impose Fickian diffusion γ = *Dq*^2^ in a global fit, i.e., simultaneous fit along *q* and ω as in previous studies,^64,70^ where *D* represents the average COM diffusion. β(*q*) is an amplitude scaling factor that accounts for the Debye–Waller factor.

For solutions containing two different types of proteins with different sizes and, thus, different diffusion coefficients, it has been shown that the scattering signal can be described by either one Lorentzian accounting for an average COM diffusion or by two Lorentzians accounting for two COM diffusion coefficients.^74^ Inspired by the latter approach, we allow for separate COM coefficients in the dilute and dense phases, denoted as *D*_dil_ and *D*_dense_, respectively, in our fits. These two values are constrained by *D*_dense_ < *D*_dil_ and connected by the intensity ratio 0 ≤ *r* ≤ 1. Therefore, adding only two fit parameters, we generalize eq 2 to
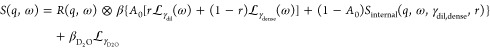
3with γ_dil_ = *D*_dil_*q*^2^ and γ_dense_ = *D*_dense_*q*^2^.

Therein, the additional dependence of *S*_internal_ on *r* and γ_dil,dense_ accounts for the separate convolution of the internal dynamics with each associated COM diffusion at the correct ratio *r*. Figure 2 depicts an example spectrum with the fit of eq 3. We emphasize that *D*_dil_ and *D*_dense_ may each account for distributions of cluster sizes represented by one effective diffusion coefficient,^70^ assuming that the two coexisting phases both show some degree of polydispersity.

**Figure 2:**
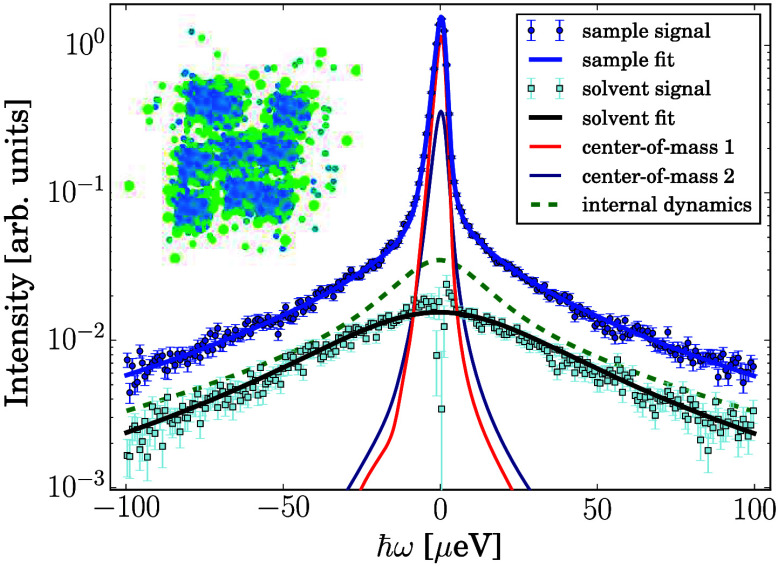
Example spectrum recorded on BSA at *c*_p_ = 240 mg/mL in D_2_O with *c*_s_ = 27 mM LaCl_3_ at *T* = 310 K and *q* = 0.55 Å^–1^ (upper circle symbols). The lower square symbols denote the corresponding pure D_2_O solvent spectrum scaled by (1 – φ). In both spectra, the container contribution was subtracted. The thick solid lines superimposed on the spectra are fits to the data. The narrow solid lines represent the Lorentzians accounting for the two separate global center-of-mass diffusion coefficients attributed to the dense and dilute phases, respectively, subsequent to the convolution with the resolution function, explaining the peculiar shape of the narrowest Lorentzian. The dashed line represents the sum of the internal diffusion of the two phases (eq 3). The inset artistic schematic illustrates the cluster distributions in the LLPS regime with local dense and dilute regions described by the Flory–Stockmayer model (cf. main text).

Depending on the best reduced χ^2^, we employ eq 2 or 3 for all samples that were not centrifuged, obtaining one or two COM diffusion coefficients *D*, depending on the sample (Figure S14). For all centrifuged samples, where the protein-rich and protein-poor phases were physically separated, we solely employ eq 2. Figure 3 represents these COM diffusion coefficients normalized to the corresponding diffusion coefficients of the BSA solution without salt at the same temperature, referred to as *D*(*c*_s_/*c*_p_, *T*)/*D*(*c*_s_/*c*_p_ = 0, *T*), illustrating the relative speeding-up of the diffusion in the dilute phase and slowing-down in the dense phase upon LLPS. The diffusion coefficients of the centrifuge-separated dense phases are very similar to those obtained for the dense phases of the corresponding mixed samples (Figure 3). This observation confirms the validity of modeling the two phases in mixed, i.e., noncentrifuged, samples by two diffusion coefficients. Furthermore, the reduced diffusion coefficients *D*(*c*_s_/*c*_p_, *T*) /*D*(*c*_s_/*c*_p_ = 0, *T*) for the dense phases decrease approximately linearly with *T*, indicating an increasing cluster size with increasing *T*.

**Figure 3:**
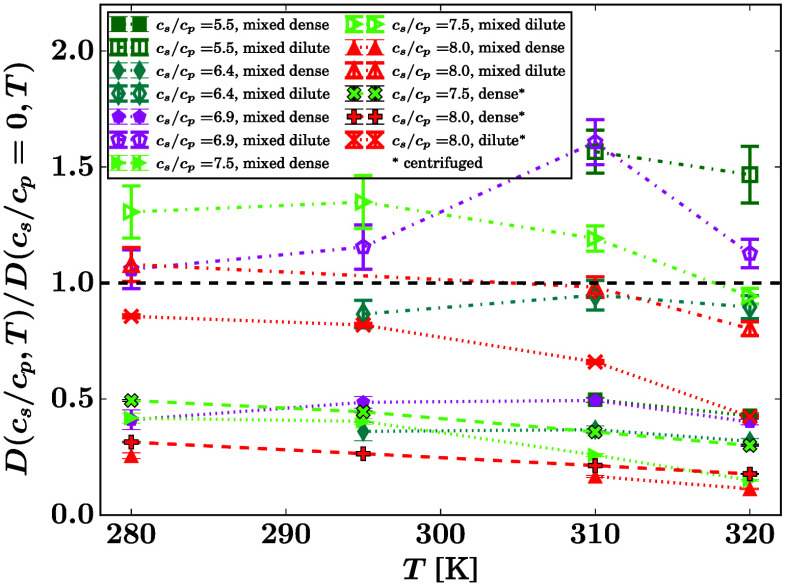
Summary of the reduced apparent center-of-mass diffusion coefficients. Depending on the salt concentration *c*_s_, temperature *T*, and centrifuging, one or two diffusion coefficients *D* were fitted. In the plot, the fit results are displayed after normalization to the diffusion coefficient in the absence of salt at the respective temperature *D*(*c*_s_/*c*_p_ = 0, *T*). The legend denotes the calculated number of salt ions per protein *c*_s_/*c*_p_ and whether the samples were centrifuged, resulting in “dense” and “dilute” phases or “mixed”. For all samples, the protein concentration as prepared was *c*_p_ = 240 mg/mL prior to centrifuging where applicable.

Figure 4 displays the ratio of the apparent diffusion coefficients in the dense and dilute phase from the QENS data (left column of the legend, area-enclosing symbols connected by solid and dashed lines) for those samples where two phases were fitted. The centrifuged sample (dashed line) is probably not separated as neatly as the *in situ* separated samples. In addition, Figure 4 displays the ratio of the theoretical monomer hard-sphere apparent diffusion coefficients calculated from the concentrations determined by UV–vis (Figure S9), measured on the same BSA batch (right column of the legend, tripod symbols connected by dotted lines). This calculation using the UV–vis determined volume fractions was carried out based on the theoretical apparent short-time diffusion coefficients of colloidal hard spheres described by scalar functions *D*_t_(φ, *T*) = *D*_0,t_(*T*)*f*_t_(φ) and *D*_r_(φ, *T*) = *D*_0,r_(*T*)*f*_r_(φ)^75,76^ for translational and rotational diffusion coefficients, respectively, from which the apparent diffusion *D* = *D*(*D*_r_, *D*_t_) = *D*_0_*f*(φ) is determined numerically involving series expansion and root finding,^68^ where *D*_0_(*T*) is the dilute-limit diffusion coefficient. The UV–vis results are plotted for values where this root finding converges.^68^ The inset of Figure 4 displays the UV–vis versus the QENS results at *T* = 303 K. The resulting symbols coincide with the bisector (line) within the error, corroborating the agreement of the QENS and UV–vis results in this picture within the limits of the centrifugation efficiency affecting the UV–vis samples.

**Figure 4:**
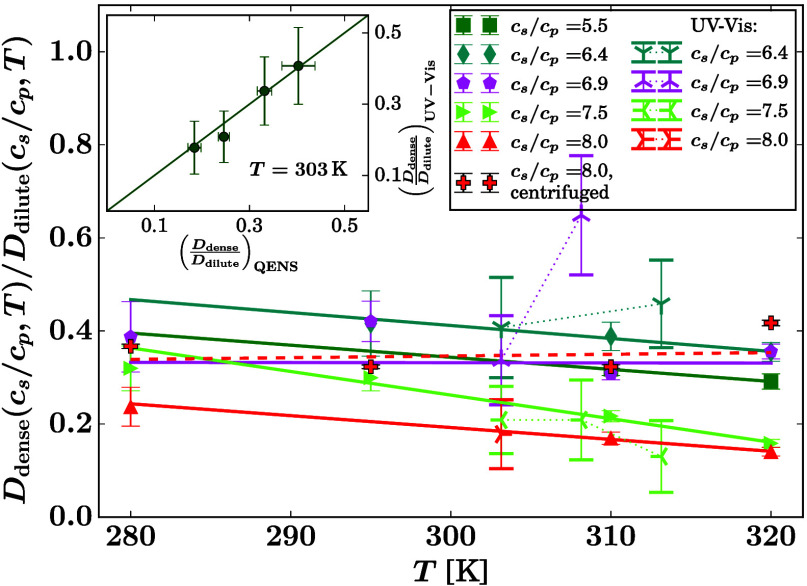
Ratio of the reduced apparent center-of-mass diffusion coefficients in the dense and dilute phase of the samples at identical *c*_s_, *c*_p_, *T* (filled symbols) and calculated ratio for the apparent diffusion of hard-sphere monomers *D* = *D*_0_*f*(φ) at the same concentrations (cf. eq 4), assuming the concentrations *c*_p_ and resulting protein volume fractions φ measured by UV–vis in the centrifuged dense and dilute phases, respectively, on the identical same protein batch at identical *c*_s_, *c*_p_, *T* (tripod symbols). The solid and dashed lines are error-weighted linear fits to the QENS results (dashed for the centrifuged sample). The dotted lines are guides for the eye for the UV–vis results. The inset reports the values at *T* = 303 K from UV–vis versus the fitted values at this temperature from QENS, from the main plot (symbols). The line marks the bisector.

We interpret the results depicted in Figures 3 and 4 as follows. Both the salt and protein concentrations in both dense and dilute phases upon LLPS change relative to the homogeneous mixture, i.e., both *c*_s_ and *c*_p_ split up in some form into local concentrations. A graphical representation of a constant-*T*-cut through the LLPS phase diagram is reported in Figure 1c. Both the dense and dilute phase should be on the phase boundary (at opposing sides), cf. the dashed line in Figure 1c.

Based on previous work,^62,63,70^ the dependencies on *D*_0_ and on the crowding and salt master curves factorize. We assume that both the Stokes–Einstein scaling *D*_0_(*T*) and percolation-limit scaling cancel out in the ratio of COM diffusion coefficients. These assumptions define the central hypothesis that at a given constant temperature
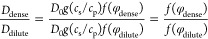
4where *f*(φ) is the scalar function describing the relative slowing-down of the COM short-time self-diffusion of monomeric colloidal hard spheres depending on the colloidal volume fraction φ, i.e., due to the crowding,^75,76^ and *g*(*c*_s_/*c*_p_) is the salt-induced master curve found earlier,^63,70^ describing the monotonous slowing-down of the self-diffusion with increasing salt concentration, depending only on the number of salt ions per protein *c*_s_/*c*_p_. This hypothesis implies that the slowing-down due to the salt-induced cluster formation cancels out. Since this slowing-down^63^
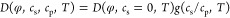
5has been found to depend only on the ratio *c*_s_/*c*_p_ at a given volume fraction and temperature, be a constant factor of 0.4 in the percolation limit,^70^ and be independent from the protein concentration after normalization to *D*(*c*_s_/*c*_p_ = 0),^70^ the simple assumption appears reasonable.

The central message combining measured LLPS COM and calculated diffusion ratios from concentrations measured by UV–vis is thus as follows: the results are consistent with the simple picture that in the LLPS regime, the cluster characteristics expressed by *g*(*c*_s_/*c*_p_, *T*) and described by the Flory–Stockmayer model^70,72,73,77^ (cf. inset of Figure 2) are the same in both the dense and dilute phase, and only the local concentration changes in the two phases. In other words, this picture implies continuity of the cluster statistics at the phase boundary. Using only the factorization of the numerator and denominator in eq 4 as input from previous work, our experiment agrees with this continuity.

There is no obvious direct association of the transition from optical transparency to turbidity (Figure 1) with any strong change in the diffusion coefficients (Figure 3). This finding indicates that although the correlation lengths in the system diverge to reach the lengths required to achieve optical turbidity, i.e., several hundred nanometers, the short-time diffusion remains consistent with small clusters independent from whether or not the sample is macroscopically separated into droplets.

The diffusion coefficients in the dense phase of the mixed samples agree very well with the diffusion coefficients in the dense phase after centrifugation (Figure 3).

From earlier work^70^ it is known that at sufficiently large *c*_s_/*c*_p_, the system can be presumed to be in the percolation limit, i.e., all bonding sites that can be involved in clustering are saturated, and the binding probability does not increase further with increasing *c*_s_/*c*_p_. Correspondingly, the distribution ρ_*n*_ of *n* clusters converges to a stable ρ_*n*_/ρ(*n*, *c*_s_/*c*_p_), with ρ being the total number density of particles, which does not increase further with increasing *c*_s_/*c*_p_.^70^ The minimum *D*/*D*_0_ in Figure 3 is similar to the minimum found for *c*_s_/*c*_p_ ≈ 9 in ref (70). Per volume and mass conservation, confirmed by microfluidic experiments,^78^ the concentrations *c* in the two phases split up according to *c*_total_ = (*c*_dense_ – *c*_dilute_)*V*_dense_^*^ + *c*_dilute_, where the normalized dense phase volume *V*_dense_^*^ = *V*_dense_/*V*_total_ is the fraction of the dense phase in the total volume. However, the volume splitting cannot be directly inferred from the QENS spectra and the fit parameter *r* of the mixed samples, since this step would require knowledge of the cluster size distribution function *g*, as opposed to the sole assumption of *g* being the same function in the two phases required for eq 4 and, thus, the comparison made in Figure 4 (cf. Figures S19 and S20).

From our QENS data, we infer that short-time diffusion is present even in the fully percolated system, i.e., the effective hydrodynamic size of the presumably transient and not necessarily compact^70^ clusters in the short-time limit does not seem to diverge, in contrast to the long-time correlation lengths observable by ultra-small-angle scattering and XPCS. This QENS observation is consistent with the finding from simulations^56^ that the system remains highly dynamic at the spinodal line. Moreover, we observe the absence of discontinuity in the temperature dependence of the diffusion when entering the optically turbid regime, suggesting that there is no (first order) phase transition in the short-time diffusion. From the possibility to fit two apparent global diffusion coefficients to the mixtures, we infer that microheterogeneity may set on much earlier than optical turbidity. This statement is corroborated by the consistent fit of distinct dense and dilute populations in the QENS spectra from the mixed phases at conditions below the associated critical temperature determined from UV–vis absorption measurements (Figure S7), which becomes obvious, for instance, for the sample depicted in Figure 1a that splits microscopically already at *T* = 280 K according to the fit (Figure S11 and Figure 3) but splits macroscopically only at higher *T* (Figure 1b).

We speculate that when small clusters that may be rigid or transient on a time scale of >1 ns cannot percolate further, the heterogeneity starts. In Figure 3, we observe that the short-time diffusion coefficient is never reduced by more than a factor of about 10 compared to that of the corresponding salt-free system. From numerical evaluations of the model cluster scattering function, we expect a maximum reduction of the salt-free diffusion coefficient by a factor of about 0.4 in the percolation limit.^70^ If the reduction of the diffusion is larger, we speculate that a deviation of the cluster size distribution from the Flory–Stockmayer model occurs.^72,73,77^ Such deviation from the Flory–Stockmayer distribution could imply that all surface charge patches on the proteins become fully “saturated” or “screened” by salt-induced charges, defining the onset of long-range correlations, possible breaking of small clusters, and dynamic exchange of proteins between clusters on a time scale beyond the coherence time of this experiment of ∼1 ns. A central question arises as to whether the system “rescales” itself such that the dense and dilute phases each keep following the corresponding master curve. In this context, we find that when we fit only one average COM diffusion coefficient, we reproduce the previous results on the master curves^70^ (Figure S16). The ratio *D*_dilute_/*D*_dense_(*T*) at constant *c*_s_ within LLPS seems to be a weakly decaying, nearly constant relation (Figure 4). In this ratio, the Stokes–Einstein dependence cancels. Thus, at a constant cluster size distribution, this ratio of diffusion coefficients represents the ratio of protein concentrations, as per the crowding effect. The trend qualitatively follows binodal separation. Our results on the short-time self-diffusion are consistent with previous findings on the different system of γ-crystallin, where LLPS was induced by crowding as the main control parameter (as opposed to charge) and where the collective diffusion was accessed, finding the absence of a discontinuity in the collective diffusion upon entering LLPS.^79,80^

We have employed spin-incoherent high-resolution QENS to access the self-dynamics of BSA in aqueous solution in the presence of a trivalent salt inducing LLPS. We stress the possible sources of ambiguity in the best choice of the model to describe these data, in particular given the complexity of this model. Notably, the possibility to fit two distinct apparent global diffusion coefficients, which each represent a distribution of clusters, does not prove that this choice of model is correct. Risks regarding the interpretation may further arise from the limited energy resolution, from an inaccurate assumption of the excluded volume in the centrifuged samples, and from other sources of uncertainty such as background scattering. Nevertheless, our results provide a simple and convincing picture of the nanosecond dynamics upon entering LLPS which, in the limiting cases explored before, is consistent with results from earlier work. We find that the short-time self-diffusion of BSA is slowed down but does not vanish at the LLPS, in agreement with the previously existing picture that protein clusters remain highly dynamic and interchanging at LLPS even in the dense phase. We present a simple hypothesis concerning the short-time self-dynamics of the transient clusters present at the LLPS, namely that while the local concentrations split in the dense and dilute phases, the short-time cluster size distribution of the presumably transient clusters in these two phases remains unchanged at the separation. Our work provides experimental evidence for this novel hypothesis by unambiguously accessing, for the first time in protein LLPS, the ensemble-averaged short-time self-dynamics informing on cluster characteristics, including systematic controls by comparing mixed and centrifuge-separated phases. It associates the results with UV–vis concentration measurements and theoretical colloid models. The new and systematic findings, employing a perfectly tunable model system, will contribute to the very active debate about the nature of LLPS.

## Experimental Methods

BSA (catalog no. A3039, batch no. SLCD4770, lyophilized powder, >98% purity, heat shock fraction, protease-free, fatty acid-free, essentially globulin-free, pH 7) and LaCl_3_ (Sigma-Aldrich, catalog no. 449830, batch no. 0000088871, anhydrous, 99.9% purity) were obtained from Sigma-Aldrich of Merck KGaA and used without additional purification. D_2_O (>99.8% purity, catalog no. 014764-30, batch no. 0438446) was obtained from Thermo Fisher Scientific (Waltham, Massachusetts, USA). Further details on the sample preparation, testing conditions, and choice of the samples for the neutron experiment are reported in the Supporting Information (SI).

The QENS experiment was carried out on the cold neutron backscattering silicon spectrometer (BASIS)^81^ at the Spallation Neutron Source (SNS), Oak Ridge, Tennessee, USA, with a pulse repetition rate of 60 Hz, employing Si(111) analyzers corresponding to an elastic wavelength of 6.27 Å, an energy resolution of ∼3.5 μeV fwhm, and an energy transfer range of −100 μeV ≤ ℏω ≤ +100 μeV, translating to the accessible time range 41 ps ≤ τ = *h*/(ℏω) ≤ 1.2 ns. With the range in momentum transfer ℏ*q*, 0.4 Å^–1^ ≤ *q* ≤ 1.9 Å^–1^, BASIS allows one to study motions at a length scale of 3.22 Å ≤ *l* ≤ 25 Å. The samples were filled into double-walled cylindrical aluminum cells (23 mm outer diameter, 0.15 mm gap between inner and outer radius), sealed against vacuum with indium wire, and inserted into a closed-cycle cryostat for the measurements. The acquisition time amounted to ∼4 h per sample per temperature.

The data were first processed using Mantid,^82^ including normalization to the incident flux and to a vanadium standard for detector calibration, and subsequently analyzed using Python, employing notably scipy.curve_fit.^66,68^ Prior to the fits, the sample container signal was subtracted, accounting for self-shielding by Paalman–Pings factors.^83^ To account for possible container batch variation, an elastic peak describing residual container scattering was, nevertheless, allowed in the fits. We write the internal dynamics in the model scattering functions (eqs 2 and 3) convoluted with the COM diffusion characterized by the line width γ as
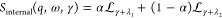
6where the sum γ + λ_1,2_ of the Lorentzian widths arises from the convolution of the internal with the COM diffusion and where the widths λ_1,2_ are coupled^71^
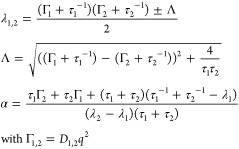
7Therein, the only free fit parameters for the internal diffusion, *D*_1,2_ and τ_1,2_, account for the internal diffusion separated into backbone and side chain motions, respectively, and are reported in the SI.

Centrifuging was performed at a constant speed of 3900 rpm (3.214 × *g*, maximum value for the selected rotor) and maintained at a temperature of 40 °C for at least 6 h until successful phase separation occurred, using an Eppendorf (Hamburg, DE) 5804-R centrifuge with a rotor radius of 18.9 cm.

Temperature-dependent UV–vis measurements were performed with a Jasco V-630 UV–vis spectrophotometer at the Partnership for Soft Condensed Matter (PSCM) in Grenoble, France, employing the identical same BSA protein batch as for the neutron experiments, starting at a set point temperature of *T* = 5 °C with temperature steps of 2.5 K and equilibration times of 15 min to observe the temperature-dependent turbidity (Figure 1). The sample temperature was recorded in the reference cell. The absorption values were normalized to the salt-free protein solution at *T*_set_ = 5 °C and averaged subsequently for the wavelength range 500 nm < λ < 700 nm.

## Data Availability

All of the code used to reduce and analyze the data as well as the UV–vis and reduced neutron data have been deposited in the ILL GitLab code repository under https://code.ill.fr/seydel/ipts-23886. Access can be granted upon request. All raw neutron data are permanently curated by the SNS under experiment number ITPS-23886.

## Supporting Information Available

Details of the LLPS testing conditions and samples, photographs of samples, UV–vis results, additional neutron data fit parameters, and estimation of phase volume ratio (PDF)
Transparent Peer Review report available (PDF)
