# Supplementary material for: Continuity of Short-Time Dynamics Crossing the Liquid–Liquid Phase Separation in Charge-Tuned Protein Solutions
Source: J Phys Chem Lett. 2024 Nov 26;15(48):12051–9. doi: 10.1021/acs.jpclett.4c02533 (PMC11756533; doi:10.1021/acs.jpclett.4c02533)
Supplement: Supplementary file 1 — jz4c02533_si_001.pdf [file jz4c02533_si_001.pdf]

Supporting Information: Continuity of short-time dynamics crossing the liquid-liquid phase separation in charge-tuned protein solutions

Ilaria Mosca[1,2], Christian Beck[1,2], Niina H Jalarvo[3], Olga Matsarskaia[2]
Felix Roosen-Runge[4], Frank Schreiber[1], and Tilo Seydel[2]

[1]*Institut für Angewandte Physik, Universität Tübingen*, Germany
[2]*Institut Max von Laue - Paul Langevin*, Grenoble, France
[3]*Neutron Scattering Division, Oak Ridge National Laboratory*, Oak Ridge, Tennessee, USA
[4]*Department of Physical Chemistry*, Lund University, Sweden

S1 Sample preparation, testing conditions for LLPS and selected samples

All preparation and testing reported in this section was carried out on site at the SNS/ORNL immediately prior to and during the neutron experiment on one single protein batch. The most challenging steps of the described experiment are sample preparation and identifying conditions for LLPS, which are described in details in the following paragraphs.

Sample preparation. BSA – bovine serum albumin – (catalog no. A3039, batch no. SLCD4770, lyophilized powder >98% purity, heat shock fraction, protease free, fatty acid free, essentially globulin free, pH 7) and $LaCl_3$ (Sigma-Aldrich catalog no. 449830, batch no. 0000088871, anhydrous, 99.9% purity) were obtained from Sigma-Aldrich of Merck KGaA and used without additional purification. D_2O ($\leq$ 99.8% purity, batch no. 0438446) was obtained from ThermoFisher Scientific (Waltham, MA, USA). A volumetric flask (VWR cat.no. 10/24-136) was used to define the liquid volumes, notably of the $LaCl_3$-D_2O stock solution. It must be noted that the location of LLPS for BSA in the (c_s, c_p, T)-space is somehow subject to batch-to-batch variability, presumably due to residual salts after the purification process [1], such that attention was paid to carry out all neutron and complementary experiments reported in this work on the identical same batch.

The samples were prepared following the protocols established in previous works [2, 3, 4]. For all samples, given amounts of BSA powder were added to D_2O and after several hours on a roller mixer at room temperature, they were all completely dissolved and the solutions appeared clear. For a chosen nominal protein concentration c_p = 240 mg/mL (corresponding to 3.613 mM), a series of samples with increasing salt concentration c_s was prepared using a 100 mM $LaCl_3$ stock solution kept in a glass volumetric flask in the fridge. As long as the salt stock solution was added to the pure BSA solutions in D_2O, they soon became milky and likely to stick on the vial walls. After 1-2 hours on the roller mixer, samples were kept for some minutes in the fridge and then left setting at lab temperature (19 ± 1)°C, ready to be tested on their behaviour against temperature variation. This step was quite easy to accomplish for the samples with low content of salt, while it was more challenging for samples

with higher c_s, which needed more time to reach a sufficiently homogeneous appearance. The 240 mg/ml BSA solutions in D_2O were prepared with the following $LaCl_3$ concentrations: $c_s = 20, 23, 25, 26, 27, 29, 30, 35$ mM.

Testing conditions for LLPS by visual inspection. Based on tests and previous works, we knew that for BSA at $c_p = 240$ mg/mL the range from 20 to 30 mM $LaCl_3$ salt concentration could be promising and started by preparing three samples at 20, 25 and 30 mM $LaCl_3$. The BSA powder was weighed and then dissolved in D_2O by keeping the tubes on a roller mixer until all visible aggregates completely disappeared. After the pure BSA solutions being ready and set, the respective amount of 100 mM $LaCl_3$ stock solution was added; this step typically causes an immediate increase of turbidity in the samples, which need some time to completely stabilize. They were therefore kept on a roller mixer for a time ranging from some hours to an entire night and later kept in the fridge for some hours until they appeared completely clear (Fig. S1).

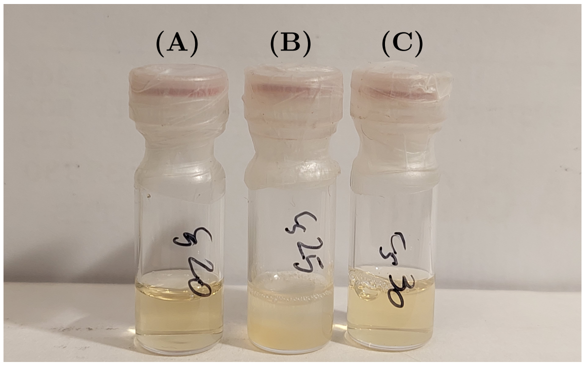

Fig. S1: BSA solutions with increasing $LaCl_3$ concentration at room temperature. A subset of the BSA solutions prepared at 240 mg/mL, with increasing concentration of the trivalent salt $LaCl_3$, namely $c_s = 20$ mM (sample **(A)**), 25 mM (sample **(B)**), 30 mM (sample **(C)**) from left to right. The picture was taken after preparing the samples and letting them set and stabilize at room temperature (18°C was the temperature reported on the thermometer of the chemistry laboratory). Just by visual inspection, one can notice that the 25 mM sample seems slightly less transparent than the two others.

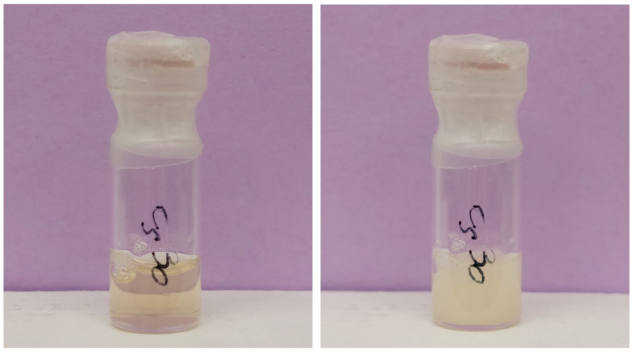

Fig. S2: BSA solution at 30 mM $LaCl_3$ after cold and hot baths. BSA solution at 240 mg/mL with 30 mM $LaCl_3$ after being for 1-2 mins in the cold bath at 0°C (left) and after 1-2 mins in the hot bath at 39-40°C (right). The sample becomes clear at 0°C, while it turns white and turbid if kept at its lower critical solution temperature (LCST). The procedure can be iterated without altering the samples, which means that the process is completely reversible.

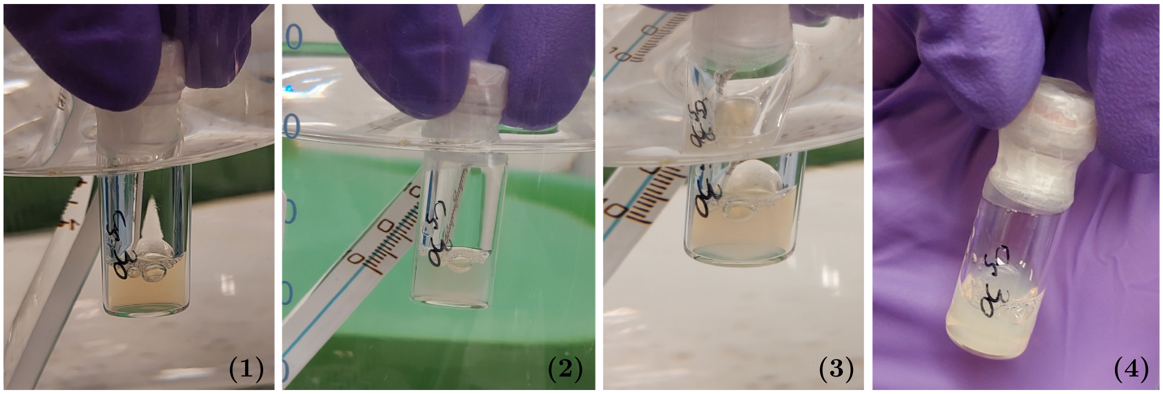

Fig. S3: Snapshots of temperature-driven changes in appearance of BSA solution with 30 mM $LaCl_3$. BSA solution at 240 mg/mL with 30 mM $LaCl_3$ **(1)** just immersed in the 39-40°C bath after being kept at 0°C, **(2)** few seconds later, **(3)** after $\sim$ 1 min and **(4)** after staying some minutes in and being removed. A transition of the sample appearance from clear/transparent to turbid/milky is observed from left to right (going from 0°C to 40°C).

In order to test the presence of LLPS, three water baths were prepared and kept at $\sim$ 0, $\sim$ 32 and $\sim$37-40°C, respectively. Each sample was immersed in the cold bath for 1-2 mins and afterwards in the hot ones. The 20 mM salt sample (Fig. S1-**(A)**) did not seem to change its appearance due to temperature variation, even if it was kept in the baths for some minutes. The other two samples (Fig. S1-**(B)**, -**(C)**) were instead more thermoresponsive, especially the one at 30 mM. After being at 0°C it became clearer than it was at lab temperature (Fig. S2, left) and then it was tested in the two hot baths: at 32°C the solution turned milky after 7-8 mins, while at 39-40°C the transition to a turbid solution only took 2 mins (Fig. S2, right). Snapshots of this transition are also reported in Fig. S3. Due to its behaviour, the 30 mM salt sample was selected as a good candidate for extracting both dense and dilute phases separately and was therefore centrifuged at 40°C overnight. In spite of showing a nice splitting between dense and dilute phases, the ratio obtained was approximately 20% dilute / 80% dense, which was not enough for the neutron experiment.

Two other sample conditions were also tested in order to look for other good candidates showing LLPS. We reached $c_s = 35$ mM and also prepared two equivalent samples at $c_s = 27$ mM, to explore the interval 25-30 mM. The 35 mM one was not very responsive to temperature variation, while the 27 mM ones showed a fast increase of solution turbidity after being immersed into the hot bath (Fig. S4). Since it exhibits strong signatures of LLPS, the 27 mM salt samples and the 25 mM one (very close in terms of c_s) were selected to extract the dense and dilute phases and were therefore centrifuged at 40°C for some hours. At the end of that step, the two 27 mM samples both showed a splitting ratio of approximately 70-65% dilute / 30-35% dense, whereas the 25 mM sample split in 85-90% dilute / 15-10% dense (Fig. S5).

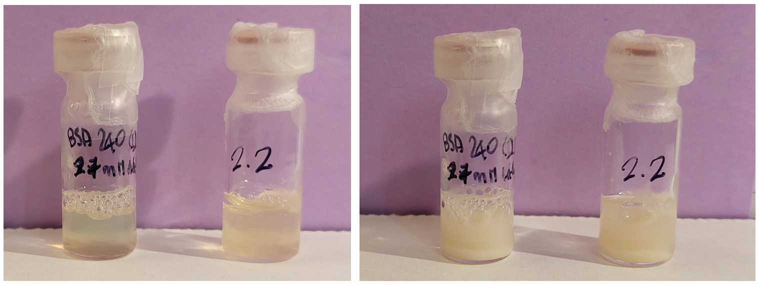

Fig. S4: BSA solutions at 27 mM $LaCl_3$ after cold and hot baths. Two equivalent samples of BSA solutions at 240 mg/mL with 27 mM $LaCl_3$ after being for around one minute in the cold bath at 0°C (left) and after one minute in the hot bath at 39-40°C (right). In the first step, the samples look clear, while after reaching the LCST and entering the phase separation regime they become milky and turbid. As above, the mechanism is reversible.

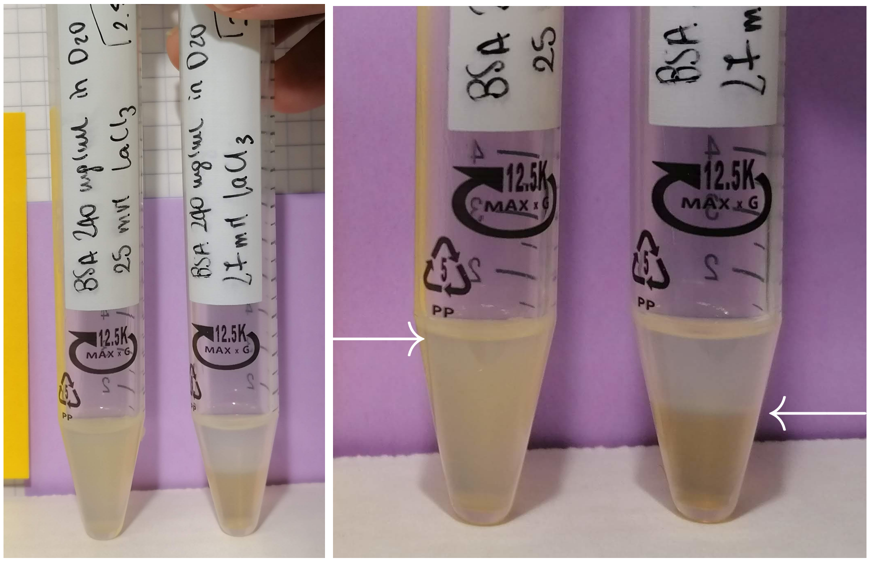

Fig. S5: BSA solutions with 27 mM $LaCl_3$ after centrifugation at 40°C. BSA solutions at 240 mg/mL with 25 and 27 mM $LaCl_3$ (respectively on the left and on the right in both images) after being centrifuged at 40°C for some hours. The figure on the right is a zoom on the sample for better visualization. White arrows indicate the meniscus / splitting interface between the dilute phase on top and the dense phase on the bottom.

Systematic search of promising samples. To test LCST-LLPS behavior on all samples prepared, two water baths kept respectively at 0°C and 37-40°C were used; temperatures were constantly monitored using immersed mercury thermometers. Each sample, contained in a glass vial or a Falcon tube, depending on the volume prepared, was immersed in the cold bath, subsequently in the hot one and then back in the cold one. By visual inspection, most samples were quite clear at the lab temperature of (19 ± 1)°C and seemed to become even clearer when placed in the cold bath ($\sim$ 0°C). When immersed in the hot bath, they turned milky and turbid after some time and became clear again when placed back in the cold bath. As mentioned before, the most promising of the prepared samples was undoubtedly the one with 27 mM $LaCl_3$ (Fig. 1 of the main article), due to its almost immediate temperature-driven change of appearance. As a consequence, the fastest changes of transparency on temperature occurred closest to c_s = 27 mM, corresponding to $c_s/c_p \approx 7.5$ salt ions per protein, as reported in Table S1. We emphasize again that the conditions found depend on the protein batch.

Mechanical separation of the phases. BSA solutions with c_s = 25, 27 and 29 mM $LaCl_3$ were centrifuged at 20000 rpm at 40°C, i.e., above LCST, during 2 to 6 hours. After centrifugation, the selected samples nicely showed a clear LLPS with different ratios of dilute and dense phase depending on their salt concentrations (Fig. 1 of the main article). All dilute phases were easily pipettable, while the dense ones were in some cases sticky, highly viscous and hard to pipette. The most promising samples in showing LLPS among the salt concentration series prepared, namely the BSA solution at c_p = 240 mg/mL with c_s = 25, 27 mM $LaCl_3$, were the ones selected to extract both dilute and dense phases arising from LLPS.

Samples measured with QENS. BSA solutions at c_p = 240 mg/mL in D_2O with $LaCl_3$ at c_s = 20, 23, 25, 27, 29 mM were measured with neutron backscattering. BSA samples with 20, 25 and 27 mM $LaCl_3$ were measured at 280, 295, 310 and 320 K in a sequence of heating steps on the same samples, and again at 280 K after cooling down for one sample to check reversibility. Dense and dilute phases extracted from the sample with c_s = 27 mM $LaCl_3$ and the dense phase from the sample with c_s = 25 mM were also measured separately, at the same temperatures and using the same heating rates. BSA c_p = 240 mg/mL + $LaCl_3$ mixtures with 23 and 29 mM $LaCl_3$ were measured at 280 K only. An empty aluminum can was measured at 295 K, along with pure D_2O at all temperatures studied, 280, 295, 310, 320 and 330 K.

$\mathbf{c_s}$ **[mM]**	0	20	23	25	27	29
$\mathbf{c_s/c_p}$	0.0	5.5	6.4	6.9	7.5	8.0

Table S1. From the salt concentration c_s, the number of salt counterions per protein c_s/c_p is calculated by assuming the nominal concentration of BSA in all samples c_p = 240 mg/mL and the molar mass of BSA m_{BSA} = 66.430 kDa. The BSA concentration given in [M] = mol/L is thus: $c_p[\mathrm{M}] = c_p[\mathrm{mg/mL}]/m_{\mathrm{BSA}}$ = 3.613 mM.

S2 Phase diagrams determined by UV-Vis spectroscopy

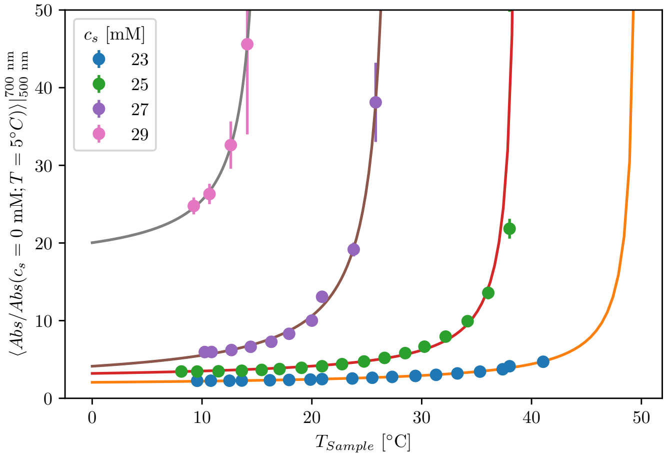

Fig. S6: Normalized averaged absorption measured with UV Vis at different temperatures for different salt concentrations as indicated in the legend. Solid lines represent the fits of the temperature dependence as explained in the main text, equation S1.

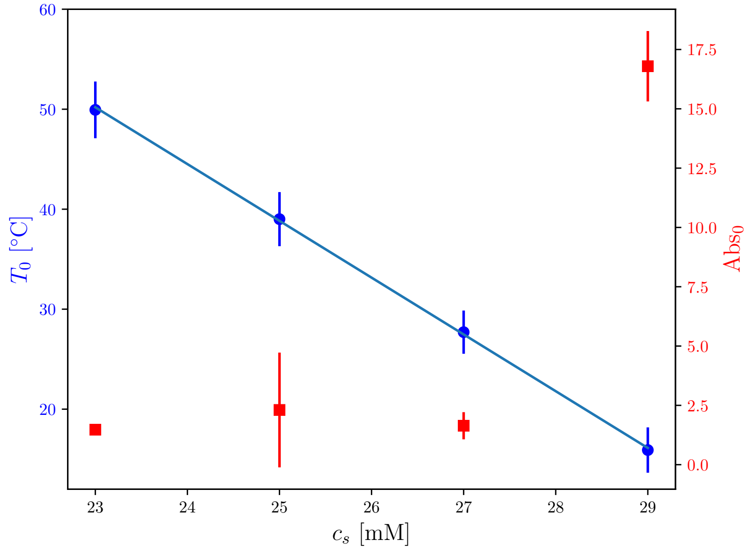

Fig. S7: Critical temperature T_0 from fits of equation S1 (figures S6) and absorption at low temperature Abs_0 as a function of c_s (symbols referring to the left and right y-axis, respectively). A linear decay can be observed in the critical temperature (blue line, slope: (-5.68 ± 0.57)°C/mM, offset 180.80 ± 15.07 °C), while an increase in the initial absorption is observed only for the highest salt concentration.

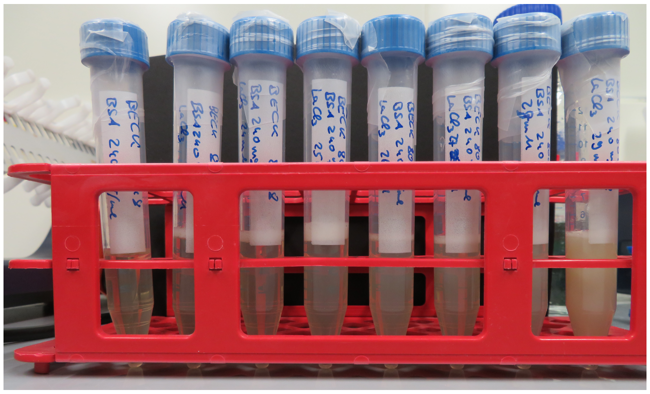

Fig. S8: Macroscopic turbidity of the samples is increasing with increasing salt concentration. Falcons shown contain $c_s = 0$ mM, 23 mM, 24 mM, 25 mM, 26 mM, 27 mM, 28 mM, 29 mM from left to right. Samples were stored at ≈ 6 °C before image acquisition.

Temperature dependent UV-Vis measurements have been performed with a Jasco V-630 UV-Vis Spectrophotometer at the Partnership for Soft Condensed Matter (PSCM) in Grenoble, employing the identical same BSA protein batch as for the neutron experiments, starting at a setpoint temperature of $T = 5$°C with temperature steps of 2.5 K and equilibration times of 15 mins to observe the temperature dependent turbidity (Fig. 1 in the main document). The sample temperature was recorded in the reference cell. The absorption values were normalized to the salt free protein solution at $T_{\mathrm{set}} = 5$°C and averaged afterwards for 500 nm $< \lambda <$ 700 nm. The temperature dependence of this average is diverging at a critical temperature T_0 which depends on c_s (Figure S6). The temperature dependence can be approximated by

$$\mathrm{Abs} = \frac{a}{T - T_0} + \mathrm{Abs}_0. \tag{S1}$$

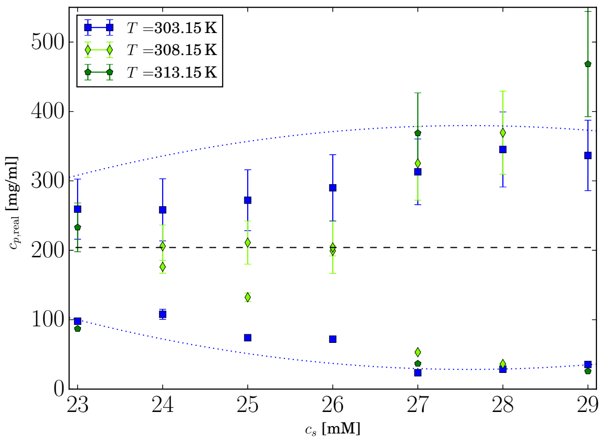

Fig. S9: Summary of UV-Vis data on the protein concentration c_p in the dense and dilute phase, respectively, versus the salt concentration c_s (symbols). The data were taken by centrifuging at 3 different temperatures, as indicated in the legend. The dashed line denotes the protein concentration at which all samples were prepared, namely at the nominal concentration $c_p = 240\,\mathrm{mg/ml}$, resulting in the real protein concentration $c_{p,\mathrm{real}} = c_p/(1 + c_p\,\nu) = 204$ mg/ml, where $\nu = 0.735$ ml/g is the specific volume of BSA. The dotted lines are guides to the eye; the lower one was obtained by a polynomial fit to the dilute concentrations measured at $T = 303.15$ K and then mirrored on the $c_p = 240$ mg/ml line to obtain the upper one.

The critical temperature T_0 decays linearly with increasing salt while the absorption Abs_0 increases with salt (Figure S7). The increase of Abs_0 does correlate nicely with the observed microscopically observed turbidity (Figure S8). To determine the protein concentration in the dense and dilute phase, the samples were centrifuged at different temperatures to separate the dense and dilute phase (Figure 2 of the main article). The volume ratio of the two phases $r = V_{\mathrm{dil}}/(V_{\mathrm{dil}} + V_{\mathrm{dense}})$ has been determined by eye. The concentration of the dilute phase has been determined by UV-Vis measurements on a dilution series performed at room temperature using the absorption at $\lambda = 280$ nm. The concentration of the dense phase has been determined using the concentration of the dilute phase, the volume ratio r as well as the mass conservation.

Based on these data, it may be attempted to describe the binodals of the phase-separation by [5]

$$T = T_0 \left(\frac{|c - c_0|}{A\, c_0} \right)^{1/\beta} + T_0 \qquad \text{(S2)}$$

with $\beta = 0.325$ corresponding to a 3D Ising system and T_0 from equation S1 (figure S6). However, since this representation would require a guess for A, it is preferable to represent the results at constant T. To this end, the concentrations in the dense and dilute phase after phase separation by centrifuging, measured by UV-Vis, are reported in Fig. S9 on samples prepared at the same aforementioned conditions and using the same protein batch as for the the neutron experiment.

The partitioning ratio should be connected to the magnitude of the second virial coefficient [6]. The densities of the phases are subject to equilibrium requirements, resulting in equal chemical potentials across the phase boundary [6, 7].

S3 Additional QENS data analysis and additional fit parameters

This section contains complementary results on internal dynamics from the QENS data fitting, along with alternative approaches used, and tests of the robustness of the analysis.

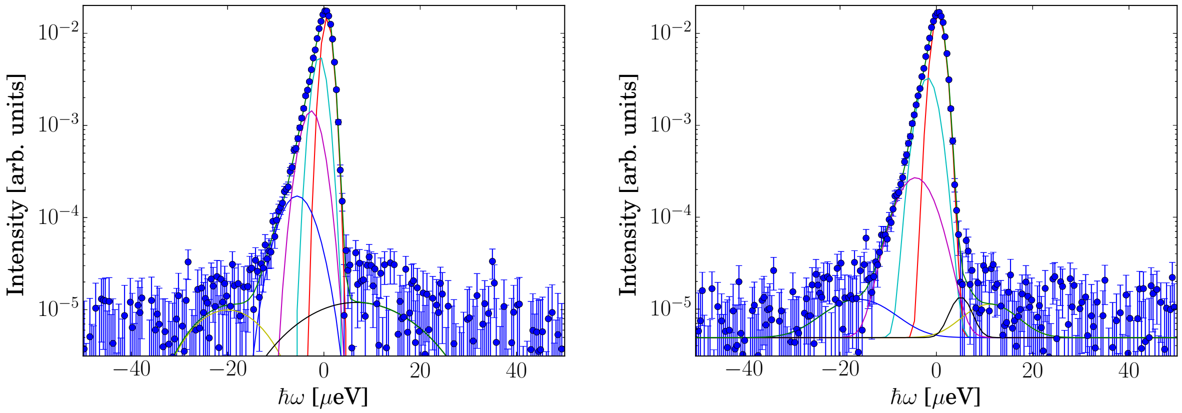

Fig. S10: BASIS spectrometer resolution function at $q = 0.75$ Å^{-1} (left) and $q = 1.25$ Å^{-1}. Energy resolution function of BASIS measured using a Vanadium foil as sample at $q = 0.75$ Å^{-1} (left) and $q = 1.25$ Å^{-1} (right, symbols) and a description by a sum of 6 Gaussian functions (lines) and a constant background. The Gaussians are used for the analytical convolution of the model function in the fits. (Note the logarithmic intensity axis, showing more than 3 orders of magnitude in accessible range, and "zoom" into the energy axis, showing only the region near the elastic peak.)

Figure S10 displays the energy resolution function of BASIS, recorded on a Vanadium foil in the same cylinder geometry as the samples, for two example momentum transfers (symbols) and its description by a sum of Gaussians functions (lines). Figure S11 displays additional example spectra recorded on a BSA solution sample, and associated fits.

Figure S12 summarizes the apparent global center-of-mass diffusion coefficients for all measured spectra during the reported QENS experiment, employing the global fits explained in the main article. Figure S13 illustrates that the data can be fitted for each q individually if the model contains not more than three Lorentizaians in total. For a larger number of Lorentzians, the fits are always carried out along the q and ω axis simultaneously in this work, consistent with previous work that has established this requirement to be able to account for more than one internal diffusive contribution [4].

Figure S14 summarizes the reduced goodness-of-fit,

$$\chi^2 := \frac{1}{N_d - N_p} \sum_{i=1}^{N_d} \frac{(y_i - f(x_i, \mathbf{p}))^2}{\sigma_i^2}, \tag{S3}$$

where y_i are the spectral intensities at points x_i along the spectral axis, f the model function evaluated at the fit parameters $\mathbf{p}$, σ_i the errors on y_i, N_d the number of data points and N_p the number of fit parameters, for all global fits in this work. From figure S14 its can be seen that the fits allowing for two separate apparent center-of-mass diffusion coefficients result in a better fit than when only allowing for one center-of-mass diffusion coefficients for non-centrifuged samples at elevated salt concentrations. The residuals reported in figure S15 confirm that the central region near $\hbar\omega = 0$ is, however slightly, better fitted with two global diffusion coefficients for samples undergoing LLPS.

Figure S16 confirms the consistency of the present with previous results [3, 8]. Figures S17, S18, and S19 summarize additional parameters from the model used to fit the QENS data described in the main article.

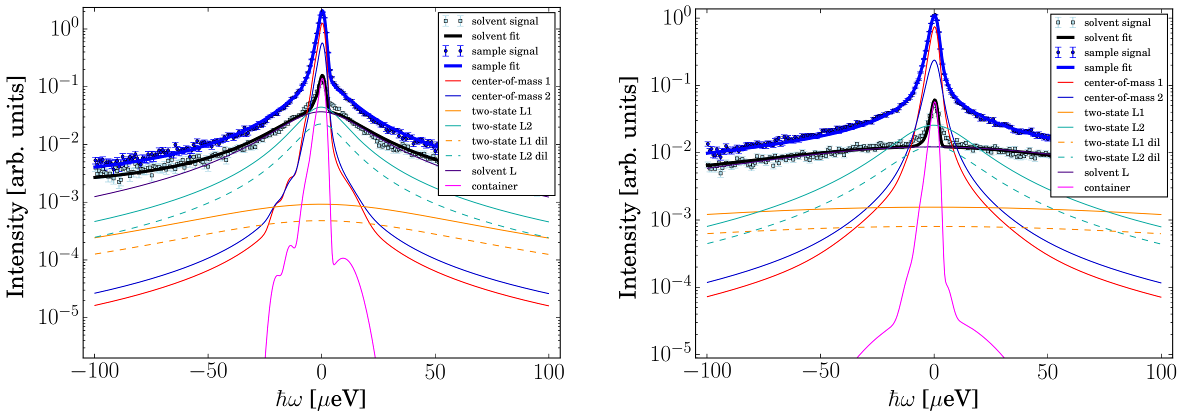

Fig. S11: Additional example spectra. QENS spectra recorded on BSA at 240 mg/ml in D_2O with 27 mM $LaCl_3$ at $T = 280$ K, at $q = 0.45$ Å^{-1} (left) and $q = 1.05$ Å^{-1} (right), respectively (upper dark circle symbols). The lower square symbols represent the D_2O solvent signal prior to the container subtraction, and the model employed here included a fit of the container signal (narrow magenta line). All fit components are shown subsequent to the convolution with the energy resolution (cf. figure S10), resulting in "skew" curves. The blue and red solid lines denote the two apparent global diffusion processes, the broader solid and dashed lines mark the coupled internal Lorentzians.

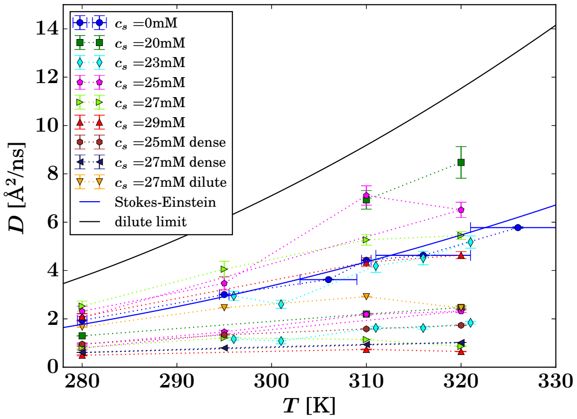

Fig. S12: Summary of the apparent center-of-mass diffusion coefficients, i.e. observable global diffusion coefficients, $D = D(D_t, D_r)$ arising from the superposition of translational D_t and rotational D_r diffusion versus temperature for the different salt concentrations explored (symbols). For $c_s = 27$ mM, the dense and dilute phases (cf. Fig. 1 in the main article), for $c_s = 29$ mM the dense phase were also measured separately after centrifuging. The upper black solid line denotes the theoretical apparent diffusion coefficient in the limit of infinite dilution $D(\varphi \longrightarrow 0)$, the lower blue solid line represents the Stokes-Einstein relation for the BSA sample without salt. The measurements on the BSA solution without salt was subject to temperature stability issues notably at the most elevated temperatures, indicated by the error bars. For all other spectra, the temperature error was smaller than the symbols.

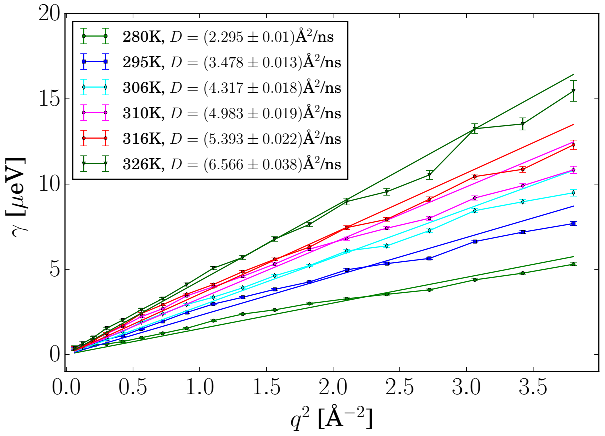

Fig. S13: Fits for each q individually of the QENS spectra of the salt-free sample. Linewidth of the first, i.e., narrowest, Lorentzian $\gamma(q)$, attributed to the apparent center-of-mass diffusion, plotted versus q^2, obtained by performing a q-wise fit of the QENS spectra of the pure BSA sample ($c_p = 240$ mg/mL), not imposing Fickian diffusion. In this case only one additional diffusion coefficient was allowed in the fit to account for the internal diffusion, as well as one solvent Lorentzian. The lines denote fits of the Fickian diffusion model $\gamma = Dq^2$. Due to the lack of a second Lorentzian accounting for internal diffusion, the thus obtained diffusion coefficients overestimate the actual diffusion.

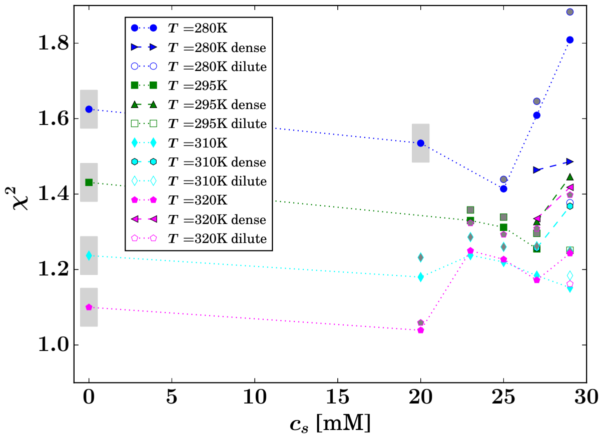

Fig. S14: Summary of the goodness-of-fit χ^2 obtained from the global fits for all samples. For the dense and dilute phases separated by a centrifuge (marked as "dense" and "dilute", respectively, in the legend) as well as for the samples marked by a grey shaded area under the respective symbol, only one center-of-mass diffusion coefficient was permitted in the fit. For the fits where two center-of-mass diffusion coefficients were employed, χ^2 for the alternative situation of one center-of-mass diffusion coefficient is in addition displayed by the corresponding grey-filled symbols with identical shape and edge color, that are always located above the associated fit with two center-of-mass diffusion coefficients. All spectra were acquired with the same integral incident neutron flux on the sample. Therefore, the systematic temperature-dependence of this error-weighted χ^2 can be attributed to the higher total spectral signal at lower T, and, thus, lower statistical error.

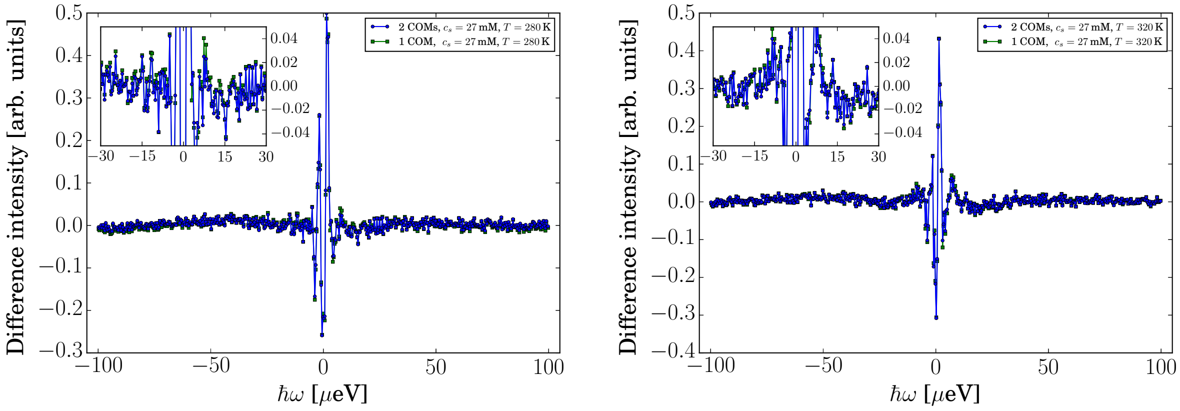

Fig. S15: Comparison of the residuals of the fits (difference between data and fit function per energy channel), summed over all q, for the model with one and two effective apparent center-of-mass diffusion coefficients (COM). Mixed sample (i.e., without external separation by centrifuging) of BSA at 240 mg/ml in D_2O with 27 mM $LaCl_3$, at $T = 280\,\mathrm{K}$ (left), and $T = 320\,\mathrm{K}$ (right). The insets represent a zoom to the main part emphasizing the region near zero energy transfer. The model with two COMs results in a very slightly but noticeably better fit (cf. figure S14).

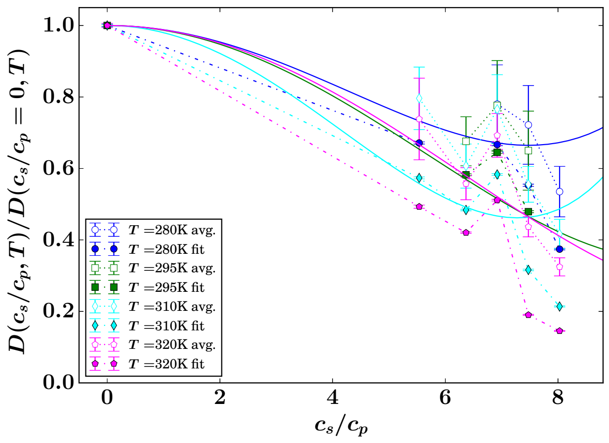

Fig. S16: Comparison of the fit result for the center-of mass diffusion coefficients (symbols) with parameterized master curves (solid lines) from previous works [8, 3]. Full symbols represent fits accounting for one single apparent center-of-mass diffusion coefficient for all samples. Open symbols represent the weighted average $[\, rD_{\mathrm{dense}} + (1-r)\, D_{\mathrm{dilute}} \,]$ for the samples where two center-of-mass diffusion coefficients were allowed. The dotted and dash-dotted lines are guides to the eye. It is emphasized that the exact location of LLPS in terms of optical turbidity strongly depends on the protein batch employed. Results from past work are, therefore, not directly comparable.

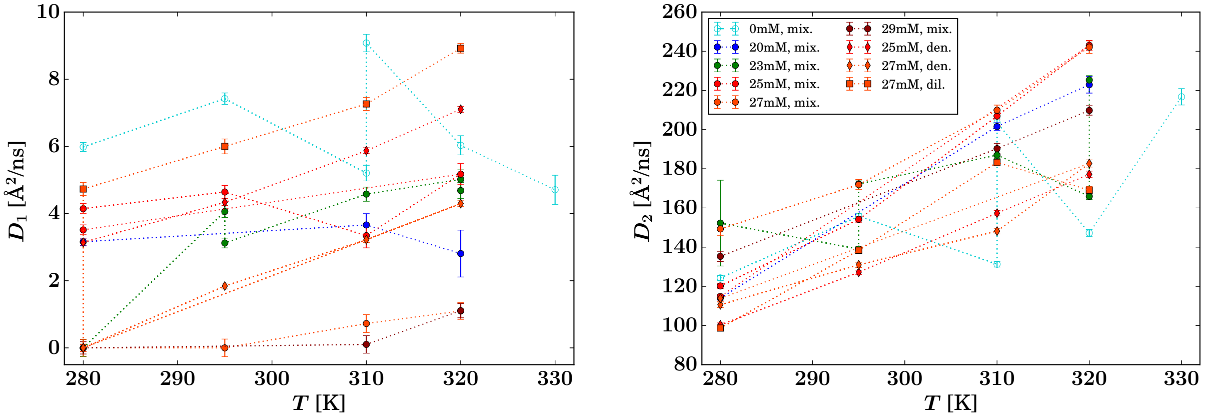

Fig. S17: Internal diffusion coefficients D_1 and D_2 of the two-diffusive states model defined in equation 5 in the main text (symbols), attributed to the protein backbone and side chain fluctuations, respectively. The lines are guides to the eye.

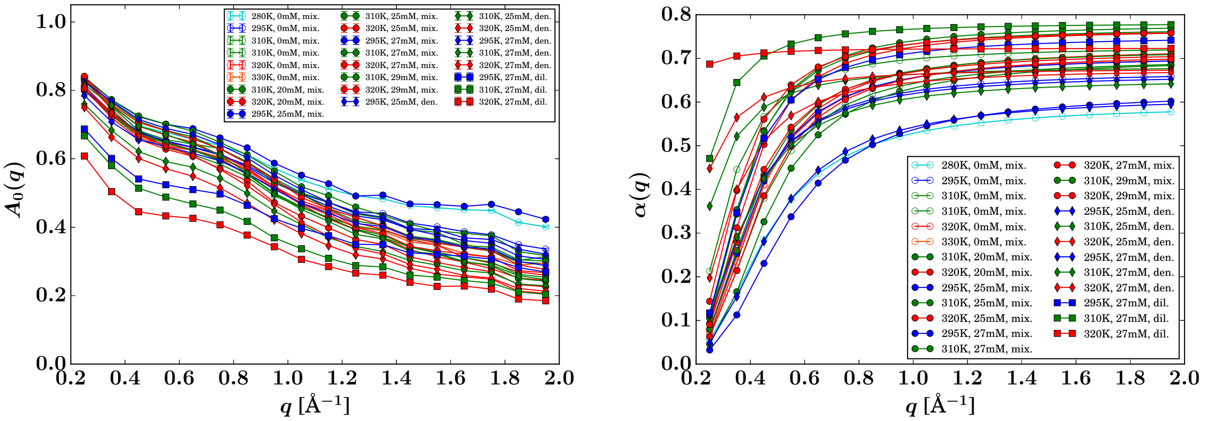

Fig. S18: Elastic incoherent structure factor $A_0(q)$ (left) and coupling parameter $\alpha(q)$ of the two diffusion processes contributing to the internal dynamics of the proteins, each with a corresponding diffusion coefficient D_1 and D_2 (cf. figure S17). Its is noted that α is not a fit parameter itself, but calculated from the model function.

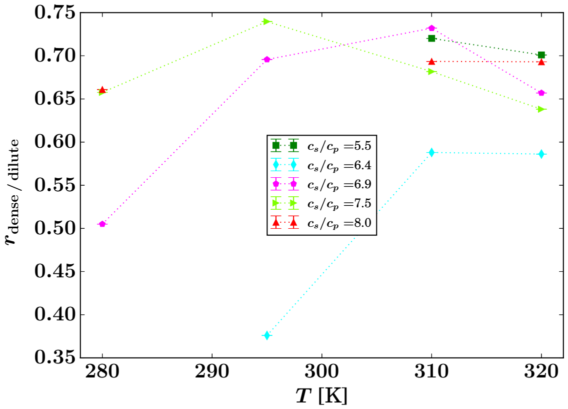

Fig. S19: Relative weight r of the apparent center-of-mass diffusion coefficient attributed to the dense and dilute phase, respectively, according to equation 5 of the main article, in the fits allowing two center-of-mass diffusion coefficients.

S4 Additional interpretation in terms of absolute volumes

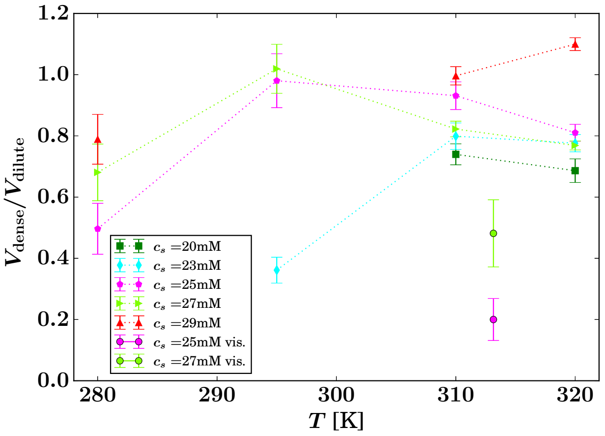

Fig. S20: Ratio of the volumes in the dense and dilute phase, respectively, calculated from the fit parameter r of the QENS spectra using equation S4 and equation S6 with $g(c_s/c_p) \equiv 1$ (symbols with edges). The circle symbols denote the ratios calculated from visual inspection.

From the implementation in the fit algorithm we have:

$$\frac{r_{\mathrm{dense/dilute}}}{1 - r_{\mathrm{dense/dilute}}} = \frac{c_{\mathrm{dense}}\, V_{\mathrm{dense}}}{c_{\mathrm{dilute}}\, V_{\mathrm{dilute}}}, \tag{S4}$$

thus,

$$c_{\mathrm{total}} V_{\mathrm{total}} = \frac{1}{1 - r_{\mathrm{dense/dilute}}} \cdot c_{\mathrm{dilute}} V_{\mathrm{dilute}}\,. \tag{S5}$$

This implementation, along with the central hypothesis from the main article, results in the following expected ratio of the splitting volumes:

$$\frac{c_{p,\mathrm{dense}}\, V_{\mathrm{dense}}}{c_{p,\mathrm{dilute}}\, V_{\mathrm{dilute}}} = \frac{\varphi_{\mathrm{dense}}\, V_{\mathrm{dense}}}{\varphi_{\mathrm{dilute}}\, V_{\mathrm{dilute}}} = \frac{f^{-1}(D_{\mathrm{dense}}\,/\,g(c_s/c_p))\, V_{\mathrm{dense}}}{f^{-1}(D_{\mathrm{dilute}}\,/\,g(c_s/c_p))\, V_{\mathrm{dilute}}}, \tag{S6}$$

where f^{-1} is the inverse of the monomeric hard-sphere colloidal crowding function $f(\varphi)$ and $g(c_s/c_p)$ the salt-induced master curve presented earlier. In contrast to equation 6 of the main article, the above equation S6 requires an explicit assumption on $g(c_s/c_p)$ in the two separated phases, since due to the inversion f^{-1}, it no longer cancels out.

An attempt to estimate the volumes in the two phases was done by visual inspection, comparing the photograph of the centrifuged sample with an empty 15 mL Falcon tube. The empty Falcon was put in front of the photograph of the BSA samples with 25 and 27 mM $LaCl_3$ ($c_s/c_p = 6.9$ and 7.5, respectively; see Fig. S5) to adjust the zoom of the latter based on the size of the real tube and then placed next to it to estimate the position of the meniscus between the dense (bottom) and the dilute phase. Using this approach, for the sample with $c_s/c_p = 7.5$ (right tube, Fig. S5) we estimated that the amount of the dense phase on the total 1 mL sample is $\sim$ 0.3-0.35 mL, resulting in $\sim$ 0.7-0.65 mL of the dilute phase and corresponding to the 30-35 and the 70-65% on the total, respectively. For the sample with $c_s/c_p = 6.9$ (left tube in Fig. S5), we estimated a volume of 0.85-0.9 mL for the dense phase and 0.1-0.15 mL for the dilute one, with corresponding percentage values of 85-90 and the 10-15%, respectively.

By inverting equation S5, the protein concentration in the dilute phase can be calculated as

$$c_{\mathrm{dilute}} = c_{\mathrm{total}}\,(1 - r_{\mathrm{dense/dilute}})\,\frac{V_{\mathrm{total}}}{V_{\mathrm{dilute}}}\,, \tag{S7}$$

where $c_{\text{total}} = 240$ mg/mL, $V_{\text{total}} = 1$ mL, V_{dilute} is estimated from the photographs and $r_{\text{dense/dilute}}$ are the values in Fig. S19. For example, we can consider the BSA sample with 27 mM $LaCl_3$. If one estimates $V_{\text{dilute}} \approx 0.65$ mL as explained above and takes $r_{\text{dense/dilute}} \approx 0.68$ from Fig. S19, $c_{\text{dilute}} \approx 240$ mg/ml$\cdot (1 - 0.68)/0.65 \approx 118$ mg/mL is obtained, corresponding to a dilution factor of ~ 2. If instead from Fig. S5 (right) one estimates a splitting in two approximately equal volumes, $c_{\text{dilute}} \approx 240$ mg/ml$\cdot (1-0.68)/0.5 \approx 153.6$ mg/ml, corresponding to a dilution by a factor of 1.56, closer to the value of 1.6 found by Da Vela et al. [9]. Results of these calculations for the 27 mM salt sample with different splitting volumes are reported in Table S2.

It has to be stressed that for Fig. S20, $g(c_s/c_p) \equiv 1$ was assumed in the above equation S6. It is at present not clear why this assumption results in reasonable volume ratios, whereas including $g(c_s/c_p)$ of the average slowing-down – which was determined for $c_s < c^*$, assuming no LLPS, i.e., a single apparent global diffusion Lorentzian – does not give reasonable results. One may speculate that the polydispersity, i.e., cluster size distribution, giving rise to $g(c_s/c_p)$, causes an average speeding up and slowing down, respectively, in the different phases, resulting in an apparent approximate compensation of the salt-induced deviation from momomeric diffusion.

$\mathbf{c_s/c_p}$	$\mathbf{V_{dense}}$ **(mL)**	$\mathbf{V_{dilute}}$ **(mL)**	$\mathbf{r_{dense/dilute}}$	$\mathbf{c_{dilute}}$ **(mg/mL)**	**dilution factor**
7.5	0.30	0.70	≈ 0.68	109.7	~ 2.19
	0.35	0.65		118.1	~ 2.03
	0.40	0.60		128	~ 1.88
	0.50	0.50		153.6	~ 1.56 [9]

Table S2. Protein concentration in the dilute phase c_{dilute} calculated using equation S7 and dilution factor $c_{\text{total}}/c_{\text{dilute}}$ for the BSA sample with 27 mM salt, assuming different splitting volumes for the two phases and $\mathrm{r}_{\text{dense/dilute}}$ taken from the fit results in Fig. S19.

References

[1] Hulse, W. L.; Gray, J.; Forbes, R. T. Evaluating the inter and intra batch variability of protein aggregation behaviour using Taylor dispersion analysis and dynamic light scattering *International journal of pharmaceutics* **2013** *453*, 351–357.

[2] Roosen-Runge, F.; Hennig, M.; Zhang, F.; Jacobs, R. M. J.; Sztucki, M.; Schober, H.; Seydel, T.; Schreiber, F. Protein self-diffusion in crowded solutions *Proceedings of the National Academy of Sciences (USA)* **2011** *108*, 11815—-11820.

[3] Grimaldo, M.; Roosen-Runge, F.; Hennig, M.; Zanini, F.; Zhang, F.; Zamponi, M.; Jalarvo, N.; Schreiber, F.; Seydel, T. Salt-Induced Universal Slowing Down of the Short-Time Self-Diffusion of a Globular Protein in Aqueous Solution *J. Phys. Chem. Lett.* **2015** *6*, 2577–2582.

[4] Grimaldo, M.; Roosen-Runge, F.; Hennig, M.; Zanini, F.; Zhang, F.; Jalarvo, N.; Zamponi, M.; Schreiber, F.; Seydel, T. Hierarchical molecular dynamics of bovine serum albumin in concentrated aqueous solution below and above thermal denaturation *Phys. Chem. Chem. Phys.* **2015** *17*, 4645–4655.

[5] Matsarskaia, O.; Braun, M. K.; Roosen-Runge, F.; Wolf, M.; Zhang, F.; Roth, R.; Schreiber, F. Cation-Induced Hydration Effects Cause Lower Critical Solution Temperature Behavior in Protein Solutions *J. Phys. Chem. B* **2016** *120*, 7731–7736.

[6] Surfaro, F.; Maier, R.; Pastryk, K.-F.; Zhang, F.; Schreiber, F.; Roth, R. An alternative approach to the osmotic second virial coefficient of protein solutions and its application to liquid-liquid phase separation *J. Chem. Phys.* **2023** *158*, 164902.

[7] Mittag, T.; Pappu, R. V. A conceptual framework for understanding phase separation and addressing open questions and challenges *Molecular cell* **2022** *82*, 2201–2214.

[8] Beck, C.; Grimaldo, M.; Braun, M. K.; Bühl, L.; Matsarskaia, O.; Jalarvo, N. H.; Zhang, F.; Roosen-Runge, F.; Schreiber, F.; Seydel, T. Temperature and salt controlled tuning of protein clusters *Soft Matter* **2021** *17*, 8506–8516.

[9] Da Vela, S.; Braun, M. K.; Dörr, A.; Greco, A.; Möller, J.; Fu, Z.; Zhang, F.; Schreiber, F. Kinetics of liquid–liquid phase separation in protein solutions exhibiting LCST phase behavior studied by time-resolved USAXS and VSANS *Soft Matter* **2016** *12*, 9334–9341.
