# Supplementary material for: Continuity of Short-Time Dynamics Crossing the Liquid–Liquid Phase Separation in Charge-Tuned Protein Solutions
Source: J Phys Chem Lett. 2024 Nov 26;15(48):12051–9. doi: 10.1021/acs.jpclett.4c02533 (PMC11756533; doi:10.1021/acs.jpclett.4c02533)
Supplement: Supplementary file 2 — jz4c02533_si_002.pdf [file jz4c02533_si_002.pdf]

2nd supplementary file: Transparent Peer Review Report

jz-2024-02533y.R1

Name: Peer Review Information for "Continuity of short-time dynamics crossing the liquid-liquid phase separation in charge-tuned protein solutions"

First Round of Reviewer Comments

Reviewer: 1

Comments to the Author

The manuscript reports neutron scattering experiments combined with UV/vis in order to study the short-time self-diffusion of a model protein-salt system undergoing liquid-liquid phase separation (LLPS). This is a very important topic and of interest to a broad community.

My impression is that the very delicate neutron (QENS) experiments have been performed and analyzed with great care. In combination with UV/vis, the exp. data convincingly support the main finding that the cluster characteristics is the same both in the dilute and dense LLPS phase, while only the local concentration changes in the two phases. This is a very important new finding and thus the paper could be of interest to many researchers concerned with LLPS.

The paper is very clearly written. I particularly like the appropriate discussion of previous works from colloid or polymer science, which strengthens the present analysis. The comprehensive supplement with supporting data is also appreciated.

I only have two minor concerns:

While information on the QENS experiments is easy to find in the paper, information on the UV-vis is a bit hidden in the SI. I suggest to briefly mention in the main text.

The centrifugation is essential for the determination of the concentration of the dense phase. However, experimental details on the centrifugation (rpm, radius, g-value, test for equilibration, etc.) are missing (or did I overlook them?). I suggest to mention such details at least in the Supporting Material.

Reviewer: 2

Comments to the Author

Authors have explored the LLPS and associated dynamics of BSA and LaCl3 system using QENS technique. I have the following observations/comments

1) Appearance of turbidity is not a confirmatory test for LLPS. I found lack of any microscopic characterization techniques to visualize the microscopic species present in the turbid solution.
2) Authors used the term "Protein Clusters" in the text. What are these clusters? I found no microscopic evidence of clusters. Authors need to characterize these clusters with microscopic techniques.
3) TOC is not meaningful. Authors should report a meaningful TOC.
4) Fig 1 a and b: Both the solution show turbidity. (b) is more turbid than (a). However, author claimed (a) is transparent. Need correction
5) Finally, the conclusions drawn from this study are well known for many LLPS systems. The novelty of this study should be mentioned clearly in the text.

Author’s Response to Peer Review Comments: please turn over to next page.

Dr. Tilo Seydel
Science Division
e-mail: seydel@ill.eu

Prof. Dr. Christy Landes
Associate Editor
The Journal of Physical Chemistry Letters
American Chemical Society
Email: landes-office@jpclett.acs.org

Manuscript ID jz-2024-02533y
Revised manuscript resubmission
"Continuity of short-time dynamics crossing the liquid-liquid phase separation in charge-tuned protein solutions"
by I. Mosca, C. Beck, N. H. Jalarvo, O. Matsarskaia, F. Roosen-Runge, F. Schreiber, and T. Seydel

Tübingen, Oak Ridge, Lund and Grenoble, October 28, 2024

Dear Professor Landes:

We thank the reviewers for the critical reading of our manuscript and their reports. We are very pleased that the first reviewer ranks our manuscript as novel and significant and recommends to publish it in The Journal of Physical Chemistry Letters subsequent to very minor revisions. Successfully addressing and in part refuting the second reviewer's concerns, we are confident that our study fulfils the criteria of novelty and urgency for publication in the The Journal of Physical Chemistry Letters, in agreement with the first review. It appears that misunderstandings regarding the central messages of our manuscript have arisen upon fast reading. We have therefore optimized the figure 1 caption and conclusions, and have added explanations on the existing knowledge of clusters in BSA-salt solutions from cited literature, for an improved stand-alone readability. Since liquid-liquid phase separation (LLPS) is at the center of an outstandingly active field of research, corroborated by the numerous recent references in high-impact journals cited in our manuscript, and since the unambiguous access to short-time self-diffusion via high-resolution neutron spectroscopy constitutes an entirely novel contribution to this field, we are convinced that the publication of our results is urgent. The message on the continuity of the short-time cluster characteristics across the phase separation urgently has to become part of the discussion on the nature of LLPS.

We have thoroughly revised our manuscript in response to both reviews, which have helped us to improve it considerably. Attached to this letter we provide an item-by-item response to the issues raised by the reviewers along with the associated changes in the manuscript. For your convenience, we also provide a version of the manuscript with the changes highlighted in color.

Sincerely,

I. Mosca, C. Beck, N. Jalarvo, O. Matsarskaia, F. Roosen-Runge, F.Schreiber, T. Seydel

1 Reviewer 1

The manuscript reports neutron scattering experiments combined with UV-Vis in order to study the short-time self-diffusion of a model protein-salt system undergoing liquid-liquid phase separation (LLPS). This is a very important topic and of interest to a broad community. My impression is that the very delicate neutron (QENS) experiments have been performed and analyzed with great care. In combination with UV-Vis, the exp. data convincingly support the main finding that the cluster characteristics is the same both in the dilute and dense LLPS phase, while only the local concentration changes in the two phases. This is a very important new finding and thus the paper could be of interest to many researchers concerned with LLPS. The paper is very clearly written. I particularly like the appropriate discussion of previous works from colloid or polymer science, which strengthens the present analysis. The comprehensive supplement with supporting data is also appreciated. I only have two minor concerns:

1. **Reviewer comment:** While information on the QENS experiments is easy to find in the paper, information on the UV-Vis is a bit hidden in the SI. I suggest to briefly mention in the main text.

   Author reply: We thank the Reviewer for the very helpful comments. We add details on UV-Vis prominently in the main article, as reported below.

   Changes in the manuscript: We mention UV-Vis and centrifuging prominently by an additional sentence at the end of the sample paragraph 'The protein solution samples were prepared ...'" in the main text: "Importantly, we measured on these samples in the mixed state and, at suitable salt concentrations, on samples that were separated into the dense and dilute phases by centrifuging, and we determined concentrations by UV-Vis (cf. Experimental Methods)." We add the detailed explanation of the UV-Vis experiment from the SI to the 'Experimental methods' section of main manuscript: "Temperature dependent UV-Vis measurements were performed with a Jasco V-630 UV-Vis Spectrophotometer at the Partnership for Soft Condensed Matter (PSCM) in Grenoble, employing the identical same BSA protein batch as for the neutron experiments, starting at a set-point temperature of $T = 5°\text{C}$ with temperature steps of 2.5 K and equilibration times of 15 minutes to observe the temperature dependent turbidity (Fig. 1). The sample temperature was recorded in the reference cell. The absorption values were normalized to the salt-free protein solution at $T_{\text{set}} = 5°\text{C}$ and averaged subsequently for the wavelength range 500 nm $< \lambda <$ 700 nm."

2. **Reviewer comment:** The centrifugation is essential for the determination of the concentration of the dense phase. However, experimental details on the centrifugation (rpm, radius, g-value, test for equilibration, etc.) are missing (or did I overlook them?). I suggest to mention such details at least in the Supporting Material.

   Author reply: We add additional information on the centrifugation as suggested.

   Changes in the manuscript: We add details on the centrifuge and the parameters employed to the 'Experimental Methods' paragraph in the main article: "Centrifuging was performed at constant speed of 3900 rpm (3.214 $\times$ g, maximum value for the selected rotor) and maintained at a temperature of 40°C for at least six hours until successful phase separation, using an Eppendorf^{TM} (Hamburg, DE) 5804-R centrifuge with a with a rotor radius of 18.9 cm."

2 Reviewer 2

Authors have explored the LLPS and associated dynamics of BSA and LaCl3 system using QENS technique. I have the following observations/comments:

1. **Reviewer comment:** Appearance of turbidity is not a confirmatory test for LLPS. I found lack of any microscopic characterization techniques to visualize the microscopic species present in the turbid solution.

   Author reply: Of course turbidity is not a confirmatory test for LLPS. This statement is part of the central message of our article, cf. item 4 below. The presence of clusters in aqueous solutions of BSA with tri-valent salts has already been shown and characterized in earlier work, e.g. Refs. [1–3] already cited in the manuscript prior to revision.

   Changes in the manuscript: We change the sentence "Aqueous solutions of BSA and multivalent salts constitute a well-investigated model system for LLPS [1–3], ..." to "Protein cluster formation has been shown and characterized for aqueous solutions of BSA and multivalent salts [2, 3], including microscopy [3–5] and light scattering [6]. These solutions constitute a well-investigated model system for LLPS at suitable salt concentrations [1–3], ..."

2. **Reviewer comment:** Authors used the term "Protein Clusters" in the text. What are these clusters? I found no microscopic evidence of clusters. Authors need to characterize these clusters with microscopic techniques.

   Author reply: Cf. the item above: The clusters result from charge-induced patchy-particle interactions as described in already cited literature. The presence of clusters has been shown and characterized in cited previous work, including small-angle X-ray and neutron scattering, light scattering, as well as at suitable conditions microscope imaging, and the incoherent QENS technique of our present work provides an unambiguous access to the cluster hydrodynamic size via the self-diffusion. In the conditions of our experiment, the clusters are too small for a microscope characterization (cf. the figure below), and only scattering techniques – notably our QENS technique – can characterize them reliably. We stress that the assumption of the Flory-Stockmayer-distribution is actually not a requirement for the conclusion of equal cluster characteristics in our manuscript, since it cancels out in equation (4).

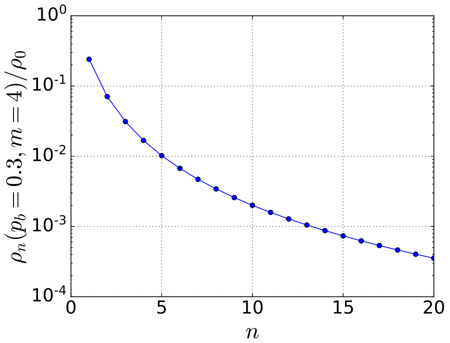

Figure 1: Normalized number density ρ_n/ρ_0 of n-clusters according to the Flory-Stockmayer theory [7–9] assuming $m = 4$ patches and a binding probability of $p_b = 0.3$, where n is the number of protein monomers per cluster.

Changes in the manuscript: We better explain the physical interpretation of S_{global} earlier in the article: We extend the sentence "..., and clusters can be represented by a single Lorentzian $\mathscr{L}_\gamma(\omega)$ with width $\gamma(q)$ accounting for the effective COM diffusion ..." to ".... It has also been shown that the clusters, which for BSA-trivalent salt solutions are distributed according to the Flory-Stockmayer theory [7–11], can be represented by a single Lorentzian $S_{\text{global}}(q,\omega) \propto \mathscr{L}_\gamma(\omega)$ with width $\gamma(q)$ accounting for the effective cluster COM diffusion ..."

3. **Reviewer comment:** TOC is not meaningful. Authors should report a meaningful TOC.

   Author reply: The ToC graphic presents a simplified artistic representation of the actual cluster size distribution based on the Flory-Stockmayer model. The left side depicts the distribution prior to LLPS, while the right side illustrates the LLPS regime with an increase in local cluster density. The ToC illustrates that the short-time cluster characteristics is unchanged by LLPS, and only the local density of clusters changes. Thus, the ToC is meaningful. We nevertheless agree that it has not been self-explanatory, so we now add labels and a colorbar for clarity.

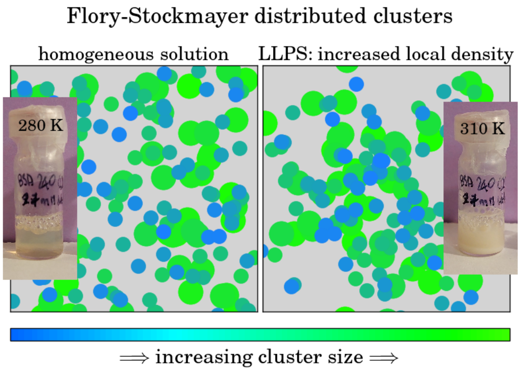

Figure 2: Revised ToC graphic

   Changes in the manuscript: To clarify, we add, "..., the cluster size distribution in each region being described by the Flory-Stockmayer model (cf. main text)" at the end of the caption of figure 2 and "(cf. schematic inset of figure 2)" behind "described by the Flory-Stockmayer model" in the main text (page 7).

4. **Reviewer comment:** Fig 1 a and b: Both the solution show turbidity. (b) is more turbid than (a). However, author claimed (a) is transparent. Need correction

   Author reply: In (a), the table and wall background is visible through the sample. By this definition, the sample is transparent. The absolute value of transparency is not the point, but change in transparency. We agree that when only reading the figure caption and conclusions without the main body of the text, the impression of a misleading definition of LLPS might arise (cf. reviewer 2 item 1 above). Therefore, we extend the figure caption.

   Changes in the manuscript: We add to the caption of figure 1: "We note that the optical transparency alone is not a suitable criterion for LLPS, cf. main text."

5. **Reviewer comment:** Finally, the conclusions drawn from this study are well known for many LLPS systems. The novelty of this study should be mentioned clearly in the text.

Author reply: Since the reviewer does not support this claim by any reference, we cannot answer to whether any of our conclusions may be "known". Per ACS guidelines, reviewers should provide "... some basis for their opinion". The literature review in our manuscript is very comprehensive and includes the most recent findings in the field. The novelty of our work lies in the first application of incoherent QENS for this system in the LLPS regime. The technique gives a unique picture of the ensemble-averaged short-time self-dynamics governed by hydrodynamic and electrostatic interactions, and an access to cluster size and to transient or short-lived clusters. Moreover, our BSA-trivalent salt model system constitutes a perfectly tunable model for LLPS, and our work provides an unprecedentedly systematic data set, resulting from an outstandingly large amount of neutron beam time on one of the spectrometers with the brightest neutron beams worldwide. The publication is urgent, because the field of LLPS research is extremely active and the finding of the continuity of the short-time cluster characteristics has to be rapidly communicated.

Changes in the manuscript: We add to the conclusions: "Our work provides experimental evidence for this novel hypothesis, by unambiguously accessing, for the first time in protein LLPS, the ensemble-averaged short-time self-dynamics informing on cluster characteristics, including systematic controls by comparing mixed and centrifuge-separated phases. It associates the results with UV-Vis concentration measurements and theoretical colloid models. The new and systematic findings, employing a perfectly tunable model system, will contribute to the very active debate on the nature of LLPS."

3 Changes requested by the editorial office

Changes in the manuscript: We have added full institution addresses for all authors.

References

[1] Matsarskaia, O.; Roosen-Runge, F.; Lotze, G.; Möller, J.; Mariani, A.; Zhang, F.; Schreiber, F. Tuning Phase Transitions of Aqueous Protein Solutions by Multivalent Cations *Phys. Chem. Chem. Phys.* **2018** *20*, 27214–27225.

[2] Matsarskaia, O.; Roosen-Runge, F.; Schreiber, F. Multivalent Ions and Biomolecules: Attempting a Comprehensive Perspective *ChemPhysChem.* **2020** *21*, 1742–1767.

[3] Saha, R.; Mitra, R. K. Thermo-Resistive Phase Behavior of Trivalent Ion-Induced Microscopic Protein-Rich Phases: Correlating with Ion-Specific Protein Hydration *Langmuir* **2023** *39*, 4601–4610.

[4] Braun, M. K.; Wolf, M.; Matsarskaia, O.; Da Vela, S.; Roosen-Runge, F.; Sztucki, M.; Roth, R.; Zhang, F.; Schreiber, F. Strong Isotope Effects on Effective Interactions and Phase Behavior in Protein Solutions in the Presence of Multivalent Ions *J. Phys. Chem. B* **2017** *121*, 1731–1739.

[5] Fries, M. R.; Conzelmann, N. F.; Günter, L.; Matsarskaia, O.; Skoda, M. W.; Jacobs, R. M.; Zhang, F.; Schreiber, F. Bulk phase behavior vs interface adsorption: Specific multivalent cation and anion effects on bsa interactions *Langmuir* **2021** *37*, 139–150.

[6] Soraruf, D.; Roosen-Runge, F.; Grimaldo, M.; Zanini, F.; Schweins, R.; Seydel, T.; Zhang, F.; Roth, R.; Oettel, M.; Schreiber, F. Protein Cluster Formation in Aqueous Solution in the Presence of Multivalent Metal Ions–a Light Scattering Study *Soft Matter* **2014** *10*, 894–902.

[7] Flory, P. J. Molecular Size Distribution in Three Dimensional Polymers. I. Gelation *J. Am. Chem. Soc.* **1941** *63*, 3083–3090.

[8] Stockmayer, W. H. Theory of Molecular Size Distribution and Gel Formation in Branched Polymers II. General Cross Linking *J. Chem. Phys.* **1944** *12*, 125–131.

[9] Roosen-Runge, F.; Zhang, F.; Schreiber, F.; Roth, R. Ion-activated Attractive Patches as a Mechanism for Controlled Protein Interactions *Scientific Reports* **2014** *4*, 1–5.

[10] Grimaldo, M.; Roosen-Runge, F.; Hennig, M.; Zanini, F.; Zhang, F.; Zamponi, M.; Jalarvo, N.; Schreiber, F.; Seydel, T. Salt-Induced Universal Slowing Down of the Short-Time Self-Diffusion of a Globular Protein in Aqueous Solution *J. Phys. Chem. Lett.* **2015** *6*, 2577–2582.

[11] Beck, C.; Grimaldo, M.; Braun, M. K.; Bühl, L.; Matsarskaia, O.; Jalarvo, N. H.; Zhang, F.; Roosen-Runge, F.; Schreiber, F.; Seydel, T. Temperature and Salt Controlled Tuning of Protein Clusters *Soft Matter* **2021** *17*, 8506–8516.
